# Influence of α‑Helical Content on the Thermodiffusion of Apomyoglobin

**DOI:** 10.1021/acs.langmuir.5c02797

**Published:** 2025-10-15

**Authors:** Binny A. Rudani, Steffen Docter, Stephan Schott-Verdugo, Johan Buitenhuis, Andreas M. Stadler, Holger Gohlke, Simone Wiegand

**Affiliations:** † Institute of Biological Information Processing IBI-4:Biomacromolecular Systems and Processes, 28334 Forschungszentrum Jülich GmbH, Jülich 52425, Germany; ‡ Institute of Bio- and Geosciences IBG-4: Bioinformatics, 9170 Forschungszentrum Jülich, Jülich 52425, Germany; § Jülich Centre for Neutron Science JCNS-1: Neutron Scattering and Soft Matter, Forschungszentrum Jülich GmbH, Jülich 52425, Germany; ∥ Institute of Physical Chemistry, RWTH Aachen University, Landoltweg 2, Aachen 52056, Germany; ⊥ Institute for Pharmaceutical and Medicinal Chemistry & Bioeconomy Science Center (BioSC), Heinrich Heine University Düsseldorf, Düsseldorf 40225, Germany; # Department für Chemie - Institut für Licht Und Materialien, Universität zu Köln, Cologne 50939, Germany

## Abstract

Apo-myoglobin (Apo-Mb) is an extensively studied model system for investigating protein folding due to its distinct stable native, partially folded molten globule (MG), and unfolded states at acidic pH. This study examines the impact of structural conformational changes on the thermodiffusive behavior of Apo-Mb using the infrared thermal diffusion forced Rayleigh scattering (TDFRS) technique. The conformational states were modulated by varying pH and buffer conditions, with their structural changes confirmed via circular dichroism (CD) spectroscopy. The α-helical content decreased with decreasing pH. The thermodiffusion parameter Δ*S*_T_(Δ*T*), a measure of the temperature sensitivity of the Soret coefficient *S*_T_, also showed a decrease, which is typically related to a decreasing hydrophilicity of the solute. Additionally, the buffer composition significantly influenced the thermodiffusive behavior: phosphate buffer promoted Apo-Mb aggregation through electrostatic screening, whereas acetate buffer favored Apo-Mb solubilization. Microsecond-long discrete protonation state constant pH molecular dynamics (CpHMD) simulations support the experimentally observed, pH- and buffer-dependent changes in α-helical content and highlight the differences in protein-buffer interactions for phosphate buffer versus acetate buffer. In conclusion, a strong correlation was observed between the thermodiffusion parameter Δ*S*_T_(Δ*T*) and the α-helical content, with Δ*S*_T_(Δ*T*) increasing alongside hydrophilicity and α-helical content. These findings highlight the role of structural conformation and buffer environment in modulating the thermodiffusive properties of proteins.

## Graphical abstract

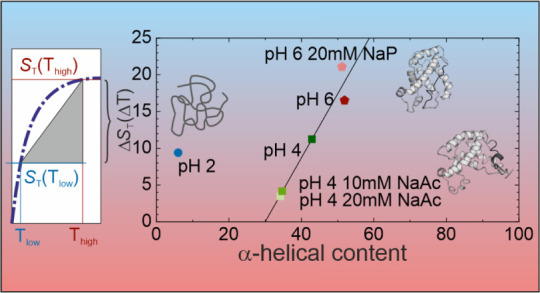

## Introduction

Thermodiffusion, first observed by Ludwig^1^ and later systematically studied by Soret,^2^ describes mass transport in a mixture driven by a temperature gradient.^3,4^ For large macromolecules and colloidal particles, the term thermophoresis is used. The physical effect provides essential information about solute–solvent interactions and is influenced by the size, charge, and structure of the solvation shell surrounding the solute molecules.^5–7^ This physical phenomenon is used in microscale thermophoresis (MST) to study biomolecular interactions, which is particularly useful for studying subtle surface changes in proteins associated with ligand binding, (un)­folding events, and other biomolecular processes.^7–10^

Thermodiffusion in a binary fluid mixture can be described as mass flux *j⃗*, which has two contributions. The first is associated with a concentration gradient −*D*∇⃗*c* and the second with a temperature gradient −*D*_T_∇⃗*T*. The two contribute to thermodiffusion in opposite ways^11^

1 $$\mathrm{j⃗}=-\rho D\mathrm{\nabla ⃗}c-\rho c(1-c)\;D_{T}\mathrm{\nabla ⃗}T$$

where *c* is the weight fraction, ρ is the mass density, and *D*_T_ and *D* are the thermal and mass diffusion coefficients, respectively. In steady state, *j⃗* = 0 and the concentration gradient induced by an applied temperature gradient is proportional to the ratio between *D*_T_ and *D*. This relationship is quantified by the Soret coefficient, *S*_T_ = *D*_T_/*D*. The sign of *S*_T_ represents the direction of mass flow: a positive sign means that the solute molecules move toward the colder region (thermophobic behavior), while a negative sign indicates that the solute moves toward the warmer region (thermophilic behavior). However, the sign of *S*_T_ is unpredictable, especially for aqueous systems, and theoretical models are still under discussion.^5,12–17^

In particular, in aqueous systems, the Soret coefficient of the solute undergoes a sign shift from negative to positive as the temperature increases.^5,5,11,18–26^ It is common to describe the thermodiffusive behavior of solutes in water by an empirical expression.^20^ In this study, the expression is presented in a modified form,

2 $$S_{T}\left(T\right)=S_{T}^{\infty }+A\;\exp\left(\frac{-T}{T_{0}}\right)$$

Here, *A* is the amplitude that measures the temperature sensitivity of *S*_T_. It is equal to $-S_{T}^{\infty }\;\exp\left(T^{*}/T_{0}\right)$ , where $S_{T}^{\infty }$ is the *S*_T_ value approached at high temperature, T* is the temperature value where *S*_T_ changes its sign, and *T*_0_ describes the bending of the curve.^20^ A measure of the temperature sensitivity of *S*_T_, the difference at two temperatures Δ*S*_T_(Δ*T*), is negatively correlated with the logarithm of the 1-octanol/water partition coefficient, log *P*.^5,23,27^ A negative log *P* indicates greater hydrophilicity of the solute.^28,29^ Thus, a highly hydrophilic solute exhibits a higher temperature sensitivity than a hydrophobic one.

However, the concept to calculate log *P*-values using heuristic or fragment-based approaches only works for relatively small molecules whose parts are always in contact with the solvent (see Section S5.3). Deviations from the correlation between Δ*S*_T_(Δ*T*) and log *P* are expected for more complex proteins. First, experimental determination of log *P* is often not possible because proteins can aggregate in pure water and require a buffer for stabilization. Second, since the hydrophilic surface accessible to the solvent depends on the folding state, a fragment-based computation for the protein’s chemical groups is highly likely incorrect. Hence, other properties must be identified to describe the hydrophilicity of the protein as a function of folding state. Systematic studies of several proteins showed that ionic strength and particle charge influence *S*_T_ less than temperature effects.^19,20^ Nevertheless, the thermodiffusive behavior of proteins is a complex interplay of surface properties, which are impacted by the ionic strength and pH of the solution.^30^

To gain deeper insights into the influence of the charge of the solute, several theoretical concepts have been developed to describe the thermodiffusion of singly charged colloidal particles.^31–38^ Both bulk and interfacial effects are considered and assumed to be additive. While the ionic shielding effect can be well described if the radius and the surface charge are known, the hydration effects have to be determined by fitting the experimental data. The theoretical concepts for colloids can only be applied to a limited extent to proteins because proteins can undergo conformational changes that may vary their hydrophilicity.

Solvent interactions significantly affect protein conformational stability by lowering energy barriers for transitions, damping collective motions, and affecting translational and rotational water entropy. Understanding hydration changes during these transitions remains challenging.^39–41^ An extensively studied model system for understanding protein folding experimentally and in molecular simulations is Apo-Mb, the heme-free form of myoglobin.^42–44^ Apo-Mb can transition between different stable conformational states with distinct structural compactness and secondary structure features.^40,41,45–48^ The globular protein consists of 153 amino acids organized into eight α-helices (A through H) connected by loops. Helix F is in an incompletely folded conformation due to the vacant heme cavity.^41,42^ The protein exhibits three primary structural states that vary with pH. At near-neutral pH (∼6), Apo-Mb adopts a compact, near native structure; at mildly acidic pH (∼4–4.5), it shifts to a partially folded *molten globule* (MG) state; at pH ∼ 2, it reaches an acid-unfolded state^49^ in the millisecond time scale.^49,50^ With decreasing pH, the net positive charge of the protein increases.^51–54^ Further details on the calculation of protein charge can be found in Sections S2 and S8. The predominantly α-helical conformation of Apo-Mb is stabilized by strong intramolecular hydrogen bonding.^55^ In an unfolded state, Apo-Mb exposes hydrophobic and hydrophilic residues, increasing water ordering around hydrophobic regions and forming structured solvation shells. These shells, driven by water’s hydrogen bonding network, occupy more volume, are less dense, and have a reduced entropy—a *hydrophobic effect*.^56^ While the hydration shells surrounding the unfolded parts of the protein enhance transient water interactions, they are less stable than in a folded state. Hydrophobic exposure reduces electrostatic interactions and effective hydrophilicity, which decreases solvation stability and promotes aggregation.^57^ Anions interact preferentially with the positively charged regions of the protein and effectively shield repulsive forces between positive charges by binding to them, thus reducing internal repulsion. In particular, anions with higher charge density and stronger affinity for the protein are more effective at inducing transitions between protein folding states than those with lower affinity.^52,58^ The stability of the *intermediate* states of Apo-Mb strongly depends on the net charge of the protein. For Apo-Mb, with a net positive charge, it has been described that the loss of positively charged residues increases stability by reducing internal charge repulsion, while the loss of negatively charged residues decreases stability due to a corresponding imbalance in charge repulsion.^59^

In molecular dynamics (MD) simulations, covalent bonds are described by force field terms, allowing variations in bond lengths and angles, but usually prohibiting bond breaking or forming of new covalent bonds. Therefore, it is common practice to determine the protonation states of titratable amino acids before starting MD simulations and then to maintain the initially set protonation states. However, in this work, effects of pH variations on Apo-Mb at the atomistic scale shall be analyzed with MD simulations. Thus, we performed discrete protonation state constant pH molecular dynamics (CpHMD) simulations in explicit solvent as implemented in the AMBER software suite.^60–62^ In this approach, the MD propagation is periodically interrupted to probe the protonation states of the predefined titratable residues via a stochastic titration method based on the Poisson–Boltzmann equation. Afterward, the protein is held fixed, while the solvent is allowed to reorganize around the newly protonated or deprotonated residues to avoid steric clashes. The MD is then further propagated for all solute and solvent constituents until the next cycle of protonation state adjustments. Applying CpHMD with Apo-Mb at different pH values allows us to investigate pH-induced effects on protein conformation and solvent interactions at picosecond resolution for microsecond-long MD simulations.

This study investigates the thermodiffusive behavior of Apo-Mb at different pH levels using the infrared thermal diffusion forced Rayleigh scattering (TDFRS) technique, highlighting hydration changes associated with the conformational states. Circular dichroism (CD) spectroscopy and CpHMD simulations confirmed changes in the predominantly α-helical structure of the protein under different conditions. The effect of acetate and phosphate buffers with different anions on Apo-Mb thermodiffusion was measured, suggesting changes in the hydration and stability of Apo-Mb. CpHMD simulations reveal changes in the protein–water hydrogen-bonding capacity and structural changes in the presence of the buffer components. Finally, correlations between α-helical content from CD spectroscopy and Soret coefficients from IR-TDFRS were explored to relate conformational to thermodiffusion properties.

## Experimental Section

### Sample Preparation

Apo-Mb was obtained from horse heart myoglobin (Sigma-Aldrich, St. Louis, MO) using the butanone extraction method to extract the heme group.^56^ The protein in the resulting solution was refolded by dialysis in 20 mM NaH_2_PO_4_/Na_2_HPO_4_ pH 7, followed by dialysis in distilled water. The refolded Apo-Mb was then lyophilized and subsequently freeze-dried. The Apo-Mb powder was then stored at −20 °C. For experimental use, a precise concentration of Apo-Mb powder was dissolved in deionized water (Milli-Q system) or buffer solutions. Specifically, 10 mM and 20 mM acetate buffer (>99.7% acetic acid, Sigma-Aldrich and >99% sodium acetate, Merck) were used for pH 4, while 20 mM NaH_2_PO_4_/Na_2_HPO_4_ (>99%, Sigma-Aldrich) buffer was used for pH 6. The acetate and phosphate buffers are abbreviated as NaAc and NaP, respectively. The clear supernatant was carefully collected after centrifugation at 29,000*g* for 10 min to remove larger aggregates. The pH of the solution was adjusted by adding 0.1 M HCl (Sigma-Aldrich). The final Apo-Mb concentration was confirmed by UV/vis absorption spectroscopy (Nano-Drop 2000c, Thermo Scientific) with an extinction coefficient (*E*_1%_) of 8.25 at 280 nm, determined from the amino acid sequence using the ExPASY web server.^63^ We characterized the protein’s secondary structure using circular dichroism (CD) and determined its thermodiffusion properties with IR-TDFRS. Additionally, we measured the electrophoretic mobility of Apo-Mb by electrophoresis. The results are summarized in Section S2.

### Circular Dichroism (CD)

CD spectra were recorded at 20 °C using a MOS-500 spectrophotometer (BioLogic, Science Instruments, France). The ellipticity of 0.9 mg/mL (∼0.05 mM) Apo-Mb, both in the presence and absence of buffer, was monitored over a wavelength range of 180 to 260 nm at different pH values in a quartz cell that is 0.1 mm thick under constant nitrogen flow. For each sample, three scans were performed and subtracted from the corresponding buffer or water values. The secondary structure content of the protein was estimated using the BeStSel single spectrum analysis software.^64^

### Thermal Diffusion Forced Rayleigh Scattering (TDFRS)

The thermal diffusion properties of the protein solution were investigated using the infrared-thermal diffusion forced Rayleigh scattering (IR-TDRFS) technique.^65,66^ An Apo-Mb solution with a concentration of 7 mg/mL (∼0.4 mM) was filled into an optical quartz cell (Hellma) with an optical path length of 0.2 mm. Measurements were performed in a temperature range from 15 °C to 45 °C in steps of 5 °C. For pH values of 6, 4, and 2, experiments were performed with and without buffer in solution. In addition, we measured both buffers at a concentration of 0.5 mol/kg. In each experiment, we collected at least 3000 individual measurement signals and calculated their average. We then examined the on and off phases of each signal, resulting in two value sets for *S*_T_ and *D*.^65,67^ Two fresh samples were measured at least twice. We therefore calculated the mean of at least four data points at each temperature. The standard deviation of the mean is shown as an error bar in the figures.

This transient grating technique uses two infrared laser beams to create a holographic grating within the sample cells. The inherent absorption of water at a wavelength of 980 nm creates a temperature grating that induces particle migration and, thus, generates a concentration gradient. The result is a refractive index grating probed by the readout laser beam. The normalized heterodyne intensity (ζ_het_(*t*)) of the readout beam, which probes the optical contrast of the interference grating, was measured and fitted to the following equation:

$$\begin{array}{l} \zeta_{\mathrm{het}}\left(t\right)=1-\exp\left(-\frac{t}{\tau_{\mathrm{th}}}\right)-A_{0}{\left(\tau -\tau_{th}\right)}^{-1}\\ \times \left\{\tau \left[1-\exp\left(-\frac{t}{\tau }\right)\right]-\tau_{\mathrm{th}}\left[1-\exp\left(-\frac{t}{\tau_{\mathrm{th}}}\right)\right]\right\} \end{array}$$ 3

where the steady-state amplitude *A*_0_ is given by

4 $$A_{0}={\left(\frac{\partial n}{\partial c}\right)}_{p,T}{\left(\frac{\partial n}{\partial T}\right)}_{p,c}^{-1}S_{T}c\left(1-c\right)$$

where τ_th_ is the heat diffusion time and τ is the mass equilibrium diffusion time. Note that the diffusion coefficient *D* and the thermal diffusivity (*D*_th_) can be derived from the corresponding equilibrium times using the relation *τ*_th_ = (*D*_th_ *q* ^2^)^−1^ and *τ* = (*Dq* ^2^)^−1^, respectively, where *q* is the scattering vector. The refractive index gradients as a function of temperature and concentration, denoted as (∂*n*/∂*T*)*_c,p_* and (∂*n*/∂*c*)*_T,p_* respectively, are measured independently (see Section S3). In addition, the Soret coefficient can be calculated from the amplitude *A* (eq 4).

### Constant pH Molecular Dynamics (CpHMD) Simulations with Discrete Protonation States in Explicit Solvent

To obtain representative structural ensembles of Apo-Mb at pH 6, 4, and 2, constant pH molecular dynamics (CpHMD) simulations with explicit solvent were performed.^61,62^ In this method, predefined titratable residues can change their protonation states upon a short Monte Carlo exchange attempt, which is relevant when considerable conformational changes or unfolding events are associated with a change in pH. The observed changes in protonation states during the simulations and the estimated p*K*_a_ values per residue are provided in Table S6. Eleven CpHMD simulation replicas were performed in five explicit solvent conditions with either water solvent with 150 mM sodium chloride, 20 mM sodium phosphate buffer (NaP), or 20 mM acetate buffer (NaAc) using the AMBER24 software suite.^60^ The entire simulation box was neutralized with respect to the positive charge of the protein by adding extra chloride ions. All simulations were based on the X-ray crystal structure of the wild-type horse heart myoglobin (PDB-ID: 2V1K). All water and buffer components and the heme and glycerol molecules from the crystal structure were removed prior to the simulation setup.

For the simulations in water solvent, the protein was placed in a truncated octahedral box of TIP3P water^68^ extending 20 Å around the protein with 150 mM of sodium chloride. The SHAKE algorithm was used to constrain hydrogen atom movements and allows 2 fs simulation time steps.^69^ Energy minimization was performed with 5,000 steps of steepest descent, followed by 5,000 steps of the conjugate gradient method, in three iterations with initial harmonic positional restraints with a force constant of 25 kcal/(mol · Å^2^) on all protein atoms, followed by a round with restraints of 5 kcal/(mol · Å^2^), and an unrestrained minimization. The minimized models were initially heated from 0 to 100 K over 10 ps using restraints of 5 kcal/(mol · Å^2^) in an NVT ensemble, followed by heating from 100 to 300 K in NPT conditions with identical restraints over 50 ps, followed by 70 ps at constant 300 K. To adjust the system density at 1 bar, 4.87 ns of unrestrained NPT simulations were performed using a Berendsen barostat,^70^ followed by a final round of 10 ns of unrestrained NVT simulations. This totals in 20.13 ns of thermalization for each of the three starting models at pH = 2, 4, and 6, respectively. Simulations containing either 20 mM of NaP or 20 mM of NaAc buffer were prepared using the PACKMOL-Memgen software.^71^ To setup cubic simulation boxes, extending 26 Å around the protein to accommodate 20 mM of the respective buffer component, using the TIP3P model^68^ for the water component. Partial charges of the buffer components were determined using RESP fitting with antechamber based on electrostatic potentials computed for optimized geometries at the Hartree–Fock 6-31G* level of theory with Gaussian09.^72^ Both simulation setups were minimized and thermalized following the protocol for the simulations in water.

The CpHMD simulations for all five equilibrated systems were carried out in 11 replicas for 3 μs each in NVT conditions. The ff10 force field^73^ was used for the protein, with the General Amber Force Field 2 (GAFF2)^74^ for the buffer components and Joung-Cheatham-parameters^75^ for sodium and chloride ions. The accumulated simulation time is 165 μs. Protonation states of all aspartate, glutamate, histidine, lysine, and tyrosine residues were determined every 5 ps, followed by 100 steps of steepest descent minimization to avoid atom clashes. In all simulations, temperature was controlled using Langevin dynamics^76^ with a friction coefficient of 1 ps^–1^ at 300 K. No barostat algorithm was used in the production runs, as they were performed in the NVT ensemble.

The CpHMD simulations were analyzed using the cphstats and cpptraj^60,77^ packages with additional in-house Python scripts. Data for simulation analyses were collected in time steps of 200 ps. The analyses used the last 500 ns of the simulations only, for which the average α-helical content became constant for the simulations at pH 6 and pH 4 (Figures S7 and S14). This measure was selected, as analysis of the conformational convergence of the simulations via RMS average correlation (RAC) plots of all backbone Cα atoms and cluster discovery analysis for both the full 3 μs trajectories, as well as for the last 500 ns (Figures S11–S13) revealed no conformational convergence over the extensive simulation time, due to the slow unfolding process of the protein and the high conformational flexibility of the MG state at pH 4.

## Results and Discussion

### Characterization of the Folding States of ApoMB

CD spectroscopy is a widely used technique to study conformational changes of proteins in solution and allows quantification of secondary structures such as α-helices, β-sheets, and random coils.^47^ However, CD spectroscopy has limitations in structural resolution and can be sensitive to environmental factors such as pH, temperature, and ligand presence, which can affect the reliability of its outcome. Compared to high-resolution techniques, such as X-ray crystallography, structural interpretations based on CD spectroscopy can be ambiguous and rely on calibration to standard data. Thus, it lacks the atomic-level detail required for in-depth structural analysis.^64,78^

The secondary structure content of Apo-Mb under different solution conditions was confirmed by CD spectroscopy. Buffers for the Apo-Mb was selected based on their p*K*_a_ values (see Section S5.1). Figure 1 shows the CD spectra of Apo-Mb in the presence and absence of buffer at different pH values. Table 1 summarizes the estimated content of α-helices under these different solution conditions, which agree with the known literature values. Note that the deviations from the literature values reach 20%, and we have found uncertainties of 3–10% in repeated measurements.

**Figure 1:**
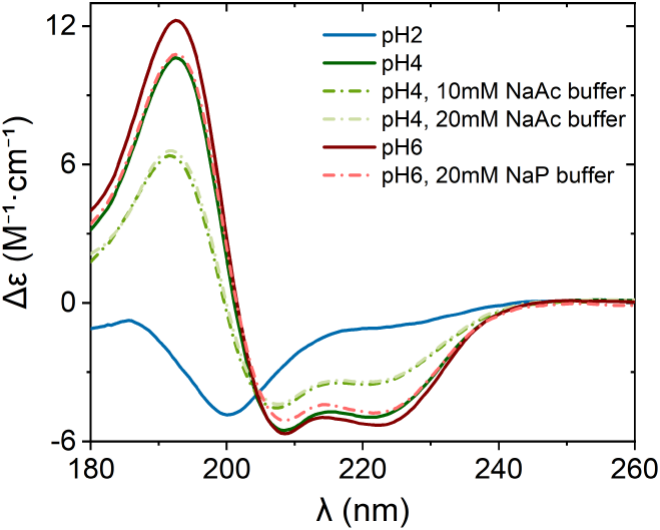
CD spectra of 52.2 μM Apo-Mb observed at 20 °C. The solid lines show Apo-Mb in water adjusted to the respective pH values with HCl, while the dashed-dotted lines represent Apo-Mb in buffer. The spectra show that the content of α-helices of Apo-Mb decreases with decreasing pH. The lines show the average of three scans.

**Table 1:** Secondary Structure Content of Different Conformational States of Apo-Mb as Measured by CD^a^

		α-helical content (%)	MD α-helical content (%)	α-helical content (%)	
protein	state	this work	this work	literature	reference
Apo-Mb at pH 2	acid unfolded	4 ± 0.3	(35)^b^	4–5	^54,82^
Apo-Mb at pH 4	MG	43 ± 2.1	42 ± 0.1	35–43	^56^,^83^ , and^84^
Apo-Mb at pH 4, 10 mM NaAc buffer	MG	33 ± 2.2	-	-	^85^
Apo-Mb at pH 4, 20 mM NaAc buffer	MG	34 ± 3.2	39 ± 0.1	-	^86^
Apo-Mb at pH 6	folded	51 ± 1.6	54 ± 0.1	49	^54^
Apo-Mb at pH 6, 20 mM NaP buffer	folded	50 ± 0.5	48 ± 0.1	55	^56^

a In the given references, the α-helical content and the state are specified. The experimentally observed α-helical content shows a strong agreement with the different conformational states defined in ref 50.

b Structure not fully unfolded within simulation time.

In the absence of buffer, CD data show that a significant fraction of α-helical structure is present in Apo-Mb at pH 6 (51%). Apo-Mb folds into a similar topology as the holoprotein at pH 6, except that helix F is incompletely folded.^41^ The content of α-helices decreases progressively with decreasing pH, from pH 6 to pH 2. At pH 6 with NaP buffer, Apo-Mb maintains its α-helical content of 50%. However, NaAc buffer at pH 4 leads to significant deviations compared to the unbuffered acidic solution and reduces the α-helical content from 43% in water to 33–34%. This indicates that NaAc buffer promotes partial unfolding of the protein in comparison to an unbuffered acidic solution. At pH 2, where the protein is in the acid-unfolded state, a small fraction of the α-helical content (4%) is retained.

To obtain atomic-level information on the structural changes upon pH changes and assess the interactions of the buffer components with the protein surface, CpHMD simulations of Apo-Mb were performed at pH 6 (with and without NaP buffer), pH 4 (with and without NaAc buffer), and pH 2, using 11 replicas per solvent condition. As the simulations are started from the holo structure, the initial helical content was higher than the values measured by CD and reached values comparable to those of the unbuffered samples after ∼2 μs for pH 6 and 2.5 μs for pH 4 (see Figures S7 and S14). Over the last 500 ns of simulation time, the average α-helical content found in the simulations overlaps with the range of experimental values determined by CD measurements for the buffered and unbuffered simulations at pH 6 and pH 4 (Figure 2).

**Figure 2:**
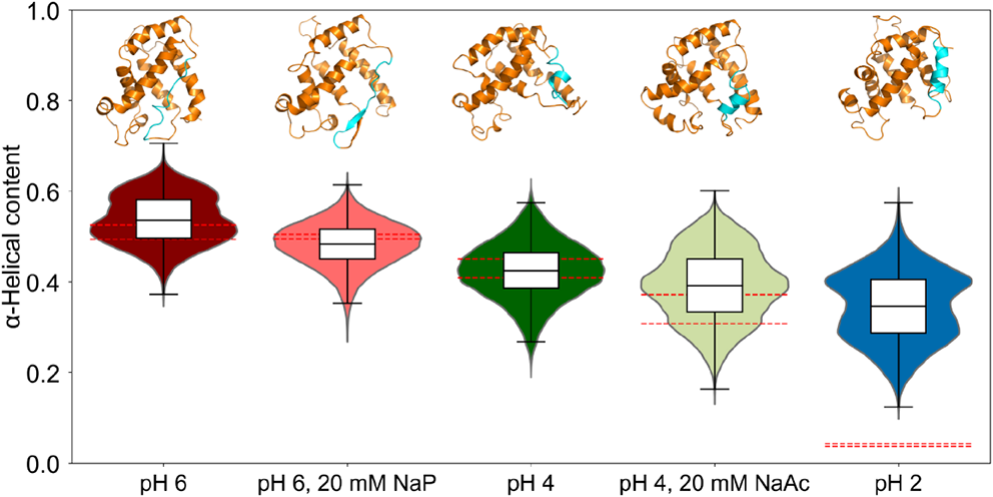
Distribution of the α-helical content of Apo-Mb over the last 500 ns of 3 μs of CpHMD simulations per replica (*n* = 11·500·5 data points, for 11 replicas, 500 ns and 200 ps of data sampling) at pH 6, 4, or 2 in explicit water, at pH 6 with 20 mM NaP buffer, or at pH 4 with 20 mM NaAc buffer. The same color scheme as in Figure 1 was used. The red dotted lines indicate the experimentally measured α-helical content ± the standard deviations (Table 1). Representative structures for each condition are shown at the top, with helix F highlighted in cyan to show the model orientations.

The distribution of the α-helical content from simulations at pH 6 in unbuffered solution is bimodal with an average of 54% ± 0.1%, with the larger peak agreeing with the experimentally measured range of 51% ± 1.6% and the smaller peak representing the higher initial α-helical content of the starting structure. By contrast, simulations with 20 mM NaP buffer at pH 6 yield a unimodal distribution around an average of 48% ± 0.1%, in agreement with the experimentally measured value of 50% ± 0.5%. At unbuffered pH 4, the distribution is unimodal around an average of 42% ± 0.1% and in close agreement with the experimental range of 43% ± 2.1%. For simulations in NaAc buffer at pH 4, the average α-helical content is 39% ± 0.1% but the variations among replicas is high, with 50% of the frames sampling conformations with an α-helical content between 33% and 45%, which encompasses the experimentally measured α-helical content of 34% ± 3.2% (Figure 2). When comparing the differences in secondary structure elements, simulations in NaAc buffer reveal a 10–20% reduction in the α-helical content for residues 16–25 in helices A and B, as well as a slight stabilization of helix F for residues 83–91. From these positions, only lysine 77 at the very end of helix E shows a significant decrease (*t*-test *p* < 0.001) in α-helical content compared to the unbuffered simulation. For the NaP simulations on the other hand, while also moderate deviations were observed, significant changes to the unbuffered simulation were only found in leucine 86 and glutamate 91 in helix F with increased α-helicality (see Figures S9 and S10). For the unbuffered simulations at pH 2, we again see large variations in the unfolding behavior between individual replicas, resulting in an average α-helical content of 35%, but find no simulation replica that samples Apo-Mb at the experimental α-helical content of 4% ± 0.3%. This is also apparent in the representative conformation, depicting the most populated structural cluster over the last 500 ns of the simulations, which shows almost no unfolding. Overall, while a marked decrease in α-helical content was observed at pH 2, also compared to the other simulations, the only partial unfolding of Apo-Mb under this condition is in line with the experimentally determined time scale on the order of (sub)­milliseconds.^50,79,80^ Yet, as suggested for Apo-Mb, a major change was observed in helix F compared to the X-ray crystal structure for Holo-Mb, which partially unfolds in the absence of the heme group in all simulated conditions.^81^

### Thermodiffusion of the Buffer

Since the temperature dependence of *S*_T_ provides some information about the hydrophilicity of the solute, we examined the NaP and NaAc buffers at a higher concentration of 0.5 mol/kg. This concentration gave a sufficiently strong heterodyne measurement signal. Figure 3 shows *D*_T_, *D*, and *S*_T_ as a function of temperature for both buffers in a temperature range between 15 °C and 45 °C. While *S*_T_ of the NaP buffer shows a strong temperature dependence, *S*_T_ of the NaAc buffer is almost temperature-independent. This is consistent with the log *P* values of −4.7 and −0.28 for NaP and NaAc, respectively,^87^ suggesting that the interaction of the buffer with the protein could have an impact on its thermodiffusion. Only the log *P* values of the major molecular buffer components in solution were considered: acetic acid for NaAc and monosodium dihydrogen phosphate for NaP (see Section S5.3). Calculating the log *P* values shows that NaP is more hydrophilic than NaAc. The temperature sensitivity of *S*_T_ of the two buffers follows the same trend as for the nonionic solutes in Figure 5 of ref 5.

**Figure 3:**
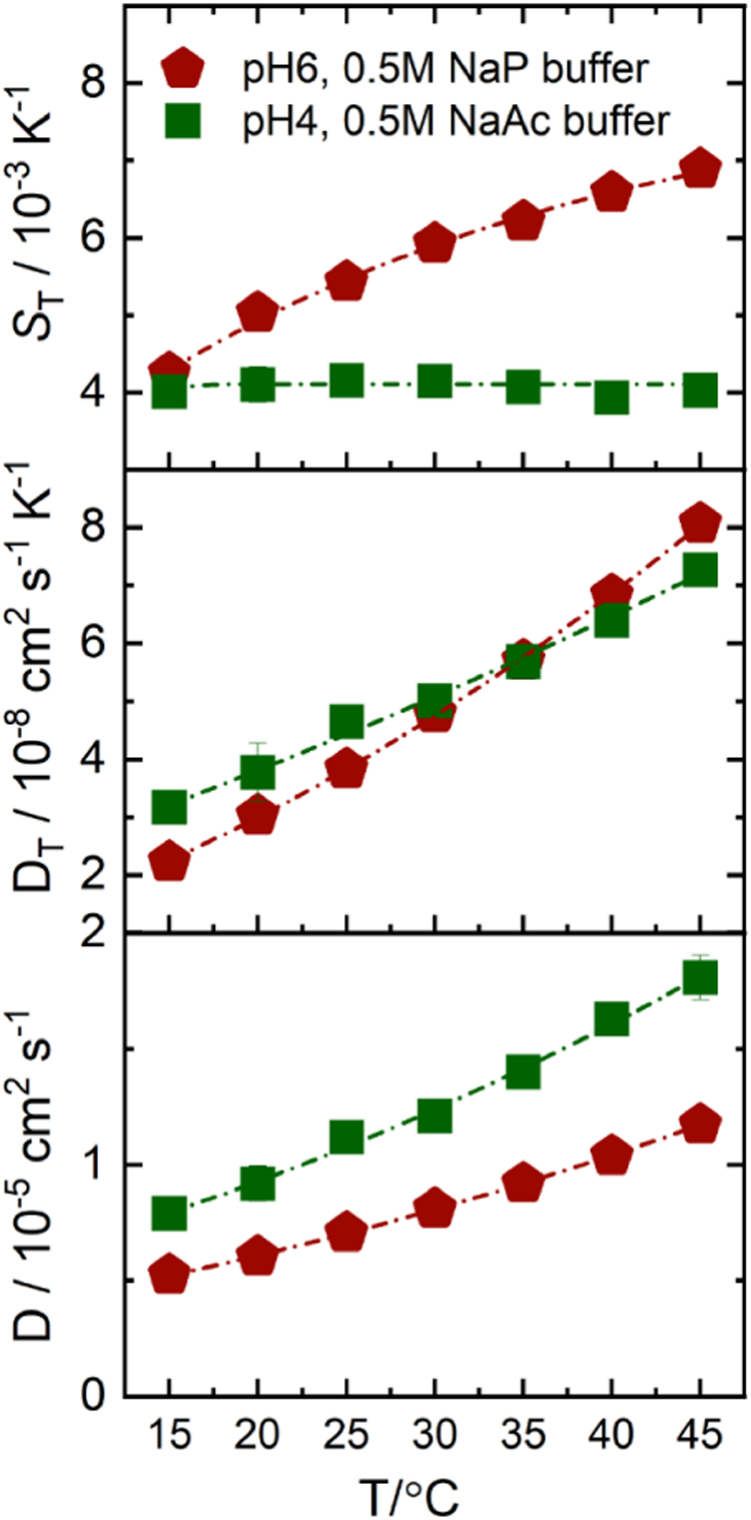
Temperature dependence of *D*_T_, *D*, and *S*_T_ of NaP (pH 6, red pentagon) and NaAc (pH 4, green square) buffers, measured at concentrations of 0.5 mol/kg. The lines are there to guide the eye. Note: For the *S*_T_ data plot, the lines are fitted according to eq 2. The fitting parameters are given in the Table S4.

Dielectric measurements also indicate a higher immobilization of water molecules in the case of NaP^88^ compared to NaAc.^89^ The NaP buffer consists of a mole fraction *x*  ≈  0.9 of monosodium dihydrogen phosphate and *x*  ≈  0.1 of disodium hydrogen phosphate. According to Eiberweiser,^88^ the number of immobilized water molecules is 4 for monosodium dihydrogen phosphate and 11 for disodium hydrogen phosphate at low concentrations. In the case of sodium acetate (*x*  ≈  0.2) and acetic acid (*x*  ≈  0.8), the number of immobilized water molecules is 5 and 1, respectively.^89^ This gives approximate values of 5.5 for NaP and 1.8 for NaAc, assuming that the mean value scales with the mole fraction *x* of the buffer components, further indicating that NaP is more hydrophilic than NaAc.

### Thermodiffusion Behavior of Apo-Mb

In the following, we discuss the thermodiffusion data of Apo-Mb in water and in buffered solutions as a function of pH. To provide a basis for understanding the thermodiffusive behavior of the protein, we first examine the IR-TDFRS results of Apo-Mb in buffer-free solutions and later in the presence of buffer. Note that the buffered Apo-Mb solutions were treated as a pseudobinary system and the contribution of the buffer to the measured signal was neglected. This is justified because we have shown that we do not observe any contribution to the concentration signal at the low buffer concentration of 20 mM (see Section S5.2 for details).

It is well-known that the diffusion properties of proteins strongly depend on their shape, size, and interaction with the environment.^40^ In recent decades, it has been experimentally demonstrated that buffer molecules can selectively adsorb onto charged protein surfaces and, thereby, influence protein–protein interactions.^90,91^ This phenomenon, which has been extensively studied in the context of simple ion adsorption on protein surfaces, is known as the Hofmeister effect.^92–94^ Previous research has shown that the precipitation of proteins by different ions is closely related to the hydration properties of the ions.^95–97^ We then examine the IR-TDFRS results in the presence of buffer compounds to assess how the buffer affects the thermal diffusion properties of the protein. This comparative approach helps to clarify to which extent buffer compounds modulate the thermodiffusive behavior of the protein compared to the unbuffered solutions.

#### Thermodiffusion of Apo-Mb at Different Unbuffered HCl-Adjusted pH-Values

Figure 4 shows the temperature dependence of *S*_T_ for Apo-Mb solutions measured at different pH values without buffer. The refractive index increments, *D*_T_ and *D* values used to calculate *S*_T_, are given in Sections S3 and S4. To minimize particle interactions, the sample concentration was kept at 0.4 mM, well below the lowest estimated overlap concentration of 9 mM, at which protein molecules begin to spatially overlap (see Section S1).^98^ The results show that Apo-Mb is thermophilic at pH 2 and pH 6 at lower temperatures, with a negative *S*_T_, but switches to a thermophobic behavior and a positive *S*_T_ when the temperature increases above ∼20 °C.^27^ In contrast, Apo-Mb remains thermophobic at pH 4 over the entire temperature range studied. However, the value of *S*_T_ increases with increasing temperature in all solution conditions and can be described by eq 2. The parameters for the different fits are listed in Table S4. The parameters $S_{T}^{\infty }$ , *A*, and *T*_0_ decrease with decreasing pH.

**Figure 4:**
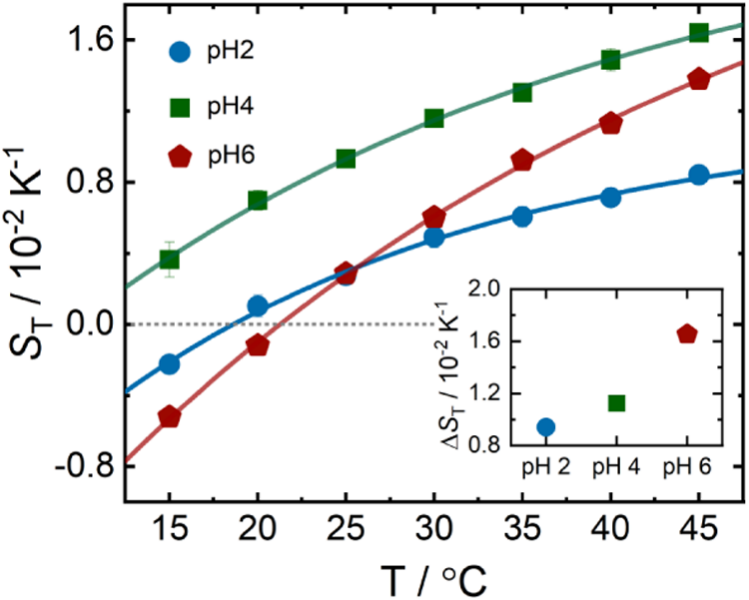
Temperature dependence of *S*_T_ of Apo-Mb without buffer at different pH (see Table 1). The solid curves represent fits to eq 2, which describes the temperature dependence of *S*_T_. The inset shows the temperature sensitivity, Δ*S*_T_(Δ*T*), as a function of pH. Note: *S*_T_(Δ*T*) refers to the difference in *S*_T_ between high (40 °C) and low (15 °C) temperatures.

Instead of using the parameter *A* in eq 2, we used the difference of *S*_T_ at a high and a low temperature and define Δ*S*_T_(Δ*T*) = *S*_T_(40 °C) – *S*_T_(15 °C) (see Section S6). The inset in Figure 4 illustrates the relationship between Δ*S*_T_(Δ*T*) and pH. Apo-Mb appears to follow the same trend as nonionic solutes, with Δ*S*_T_(Δ*T*) decreasing with decreasing pH.

A similar temperature dependence of *S*_T_ with a transition from thermophilic to thermophobic behavior with increasing temperature has also been observed for proteins such as lysozyme, β-lactoglobulin, and streptavidin.^19,22,38^ All these proteins show a positive slope for *S*_T_, with an order of magnitude of 10^–2^ K^–1^.^24,99,100^ It is postulated that hydrophobic interactions play an important role in the thermophoresis of proteins.^8,22^ In general, at high temperatures, water interacts preferentially with the charged and polar regions of proteins, promoting thermophobic behavior (positive *S*_T_). However, as temperature decreases, the hydrophobic effects become stronger, causing proteins to exhibit thermophilic behavior by favoring warmer regions.^19,101^ Unfortunately, there is no theory to predict the temperature at which *S*_T_ changes sign. Experimental results suggest that the chemical nature of the proteins and the structural properties of water contribute in a complex way.

A log *P* value cannot be determined for proteins, neither by calculation nor by experiment. However, for Apo-Mb, the pH value appears to be a reliable indicator of hydrophilicity. Apo-Mb, with an isoelectric point of 7.2, is nearly neutral at pH 6.^54^ At this pH, proteins typically adopt a folded conformation in which most of their hydrophobic regions are buried in the core and most of their hydrophilic or charged regions are exposed to the aqueous environment, so they behave like a hydrophilic molecule in solution.^39,102,103^ As the pH decreases, the protonation of the amino acids increases the net positive charge of the protein, resulting in greater electrostatic repulsion between the protein side chains. Negatively charged residues at the protein surface get neutralized, generating neutral surface patches, and reducing the interactions with water molecules. This leads to unfolding of the protein and exposure of the hydrophobic regions to the solvent.^56,104^ Indeed, in MD simulations of Apo-Mb, the number of hydrogen bonds formed between the protein and water molecules decreases with decreasing pH, whereas the solvent accessible surface area (SASA) increases and the number of intramolecular hydrogen-bonds decreases (Figure 7), in line with a denaturation process taking place due to the pH change. Consequently, the hydrophilicity of the protein decreases when the pH is lowered due to its unfolding. However, at very low pH values (e.g., pH 2), the protein attains a high net positive charge, which can enhance electrostatic hydration, while simultaneously exposing hydrophobic regions due to unfolding. The interplay between these opposing effects, electrostatic hydration and hydrophobic exposure, collectively determines the apparent hydrophilicity of the protein under such acidic conditions. Note that the process of Apo-Mb unfolding is reversible, and the protein can be easily refolded from the state of acidic denaturation.^41^

#### Influence of Phosphate Buffer at pH 6

Next, we investigated the thermodiffusion behavior of Apo-Mb specifically at pH 6. The temperature dependence of *S*_T_, *D*_T_, and *D* with and without NaP buffer is shown in Figure 5.

**Figure 5:**
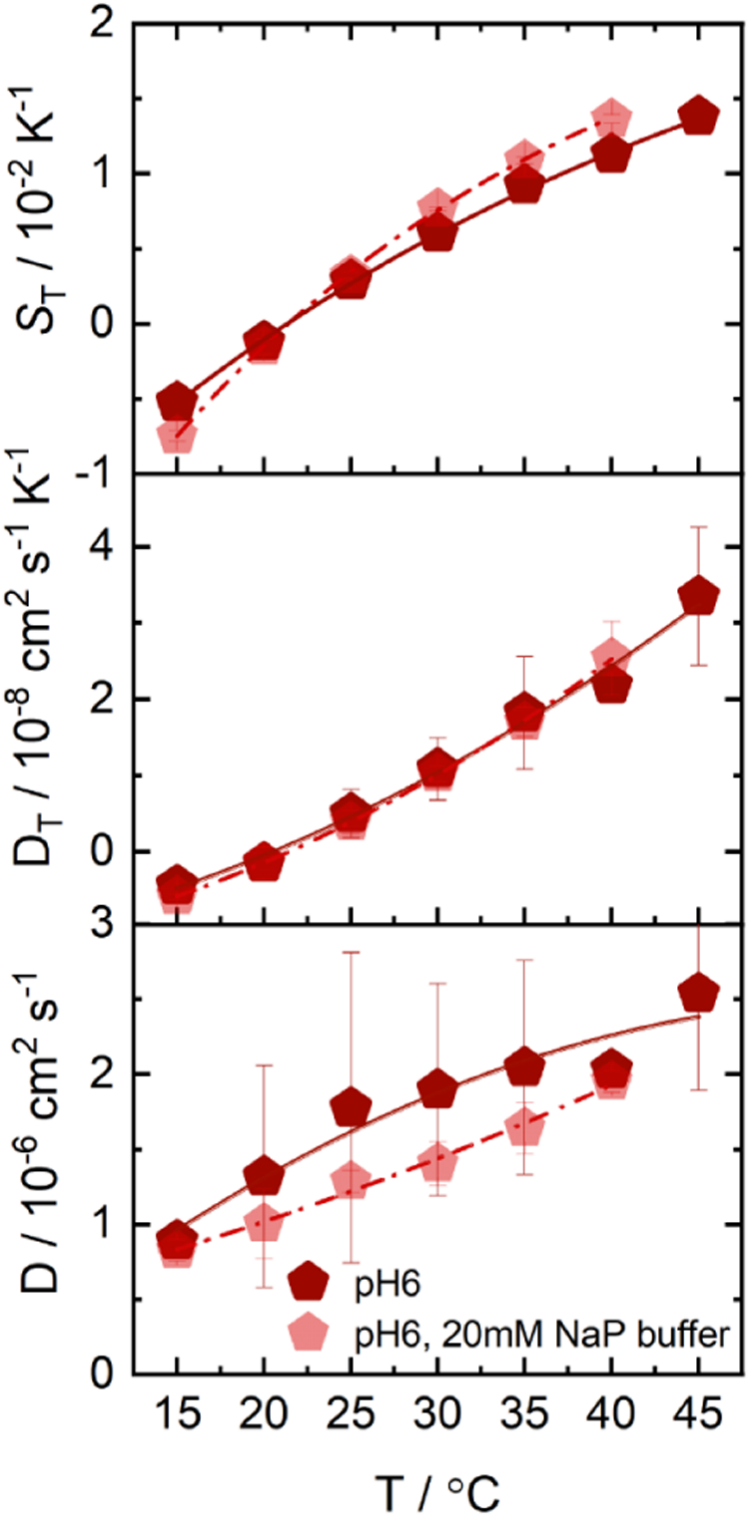
Temperature dependence of *S*_T_, *D*_T_, and *D* of Apo-Mb with and without buffer, measured at pH 6. The dark red pentagons represent the Apo-Mb solutions without buffer, adjusted with hydrochloric acid, as shown in Figure 4, while the light red pentagons correspond to Apo-Mb in NaP buffer. The curves for *S*_T_ are fitted according to eq 2 (see Table S4), while the lines for *D* and *D*_T_ are a guide for the eye.

The *D*_T_ values for Apo-Mb with and without buffer overlap over the entire temperature range, indicating that the buffer has no significant effect on the thermodiffusion behavior. This is consistent with CD measurements and the MD simulations, where Apo-Mb retains its α-helical and overall secondary structure unchanged under these conditions. In contrast, the *D* value for Apo-Mb was significantly lower in the presence of NaP buffer than in the unbuffered solutions. This decrease in *D* is mainly attributed to protein aggregation by the NaP buffer: in the presence of buffer, the light scattering intensity at 45 °C increased instantaneously, resulting in increased turbidity, making measurements impossible and indicating protein aggregation. Similar aggregation phenomena have been observed for other proteins in the presence of phosphate buffer.^58,90,105–107^ According to the Hofmeister series, the phosphate ions (e.g., ${\mathrm{HPO}}_{4}^{2-}$ or ${\mathrm{PO}}_{4}^{3-}$ ) are classified as strong kosmotropes, which are known to enhance the structuring of water molecules in their vicinity.^92^ This kosmotropic behavior can lead to protein compaction by stabilizing intramolecular interactions and reducing the exposure of hydrophobic residues to the water. Consequently, the solvent-accessible surface of the protein becomes more hydrophilic. However, phosphate ions can also promote protein aggregation due to charge neutralization under specific conditions as shown in our study. For more information on the effect of buffers on the protein, see Section S5.4. However, the effects of these buffer ions on protein aggregation can be better understood by identifying the binding site on the protein surface, which has only been discussed on an empirical basis.^107^

In CpHMD simulations, aggregation of the protein is not evaluated, as only a single Apo-Mb molecule is simulated. Still, at 20 mM NaP pH 6, the CpHMD simulations reveal that the phosphate ions bind close to and at the vacant heme cavity and at dedicated positions over the protein surface (Figure 6). This could contribute to a difference in the particle diffusion *D*, as the apparent molecule size increases compared to the unbuffered solution. Interestingly, while the protein has a similar amount of α-helices when buffered with NaP, it displays an increase in the measured SASA when compared with the unbuffered simulations. In addition, as positions on the surface of the protein are occupied by buffer molecules, the number of hydrogen bonds between the protein and water gets reduced compared to the unbuffered solvent, as well as the intramolecular hydrogen bonds of the protein (Figure 7). One has to consider that only direct hydrogen bonds with water are counted as protein–water hydrogen bonds, excluding potential cases where phosphate ions could bridge specific hydrogen bonds between the protein and water. Importantly, the main peak in the hydrogen-bond distribution with NaP shows a slight shift toward more hydrogen bonds compared to the unbuffered simulations, indicating that NaP fosters the formation of protein–water interactions in frames with no direct phosphate-protein interactions. This aligns well with the kosmotropic effect of phosphate ions, fostering protein hydrophilicity as described above.

**Figure 6:**
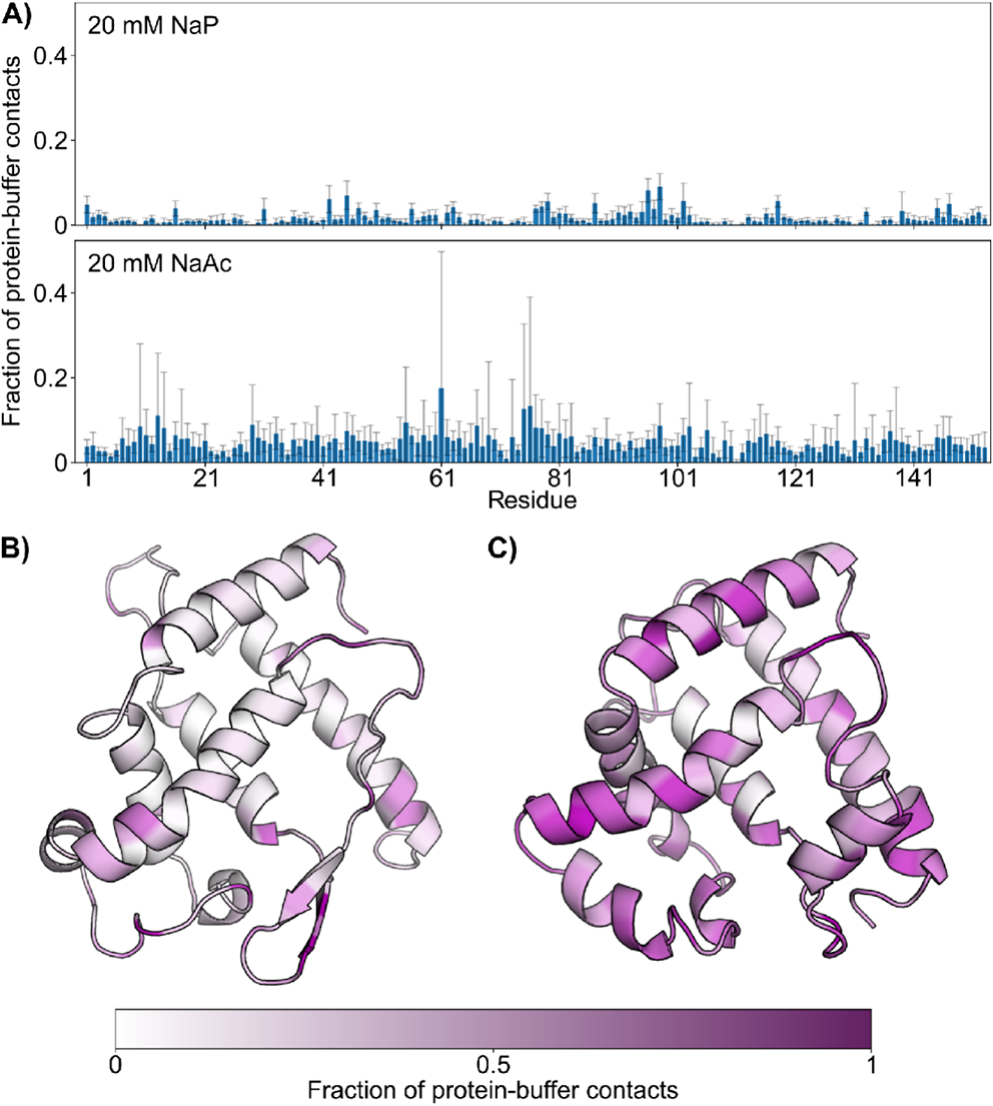
Residue-specific buffer interactions with Apo-Mb determined from CpHMD simulations. (A) Per residue average fraction of contacts between Apo-Mb and NaP (top) or NaAc (bottom) buffer molecules. (B, C) Values from A shown on representative protein structures obtained from CpHMD simulations with either 20 mM NaP (left) or NaAc buffer (right). Values are averages ± standard deviation over 11 replicas each.

**Figure 7:**
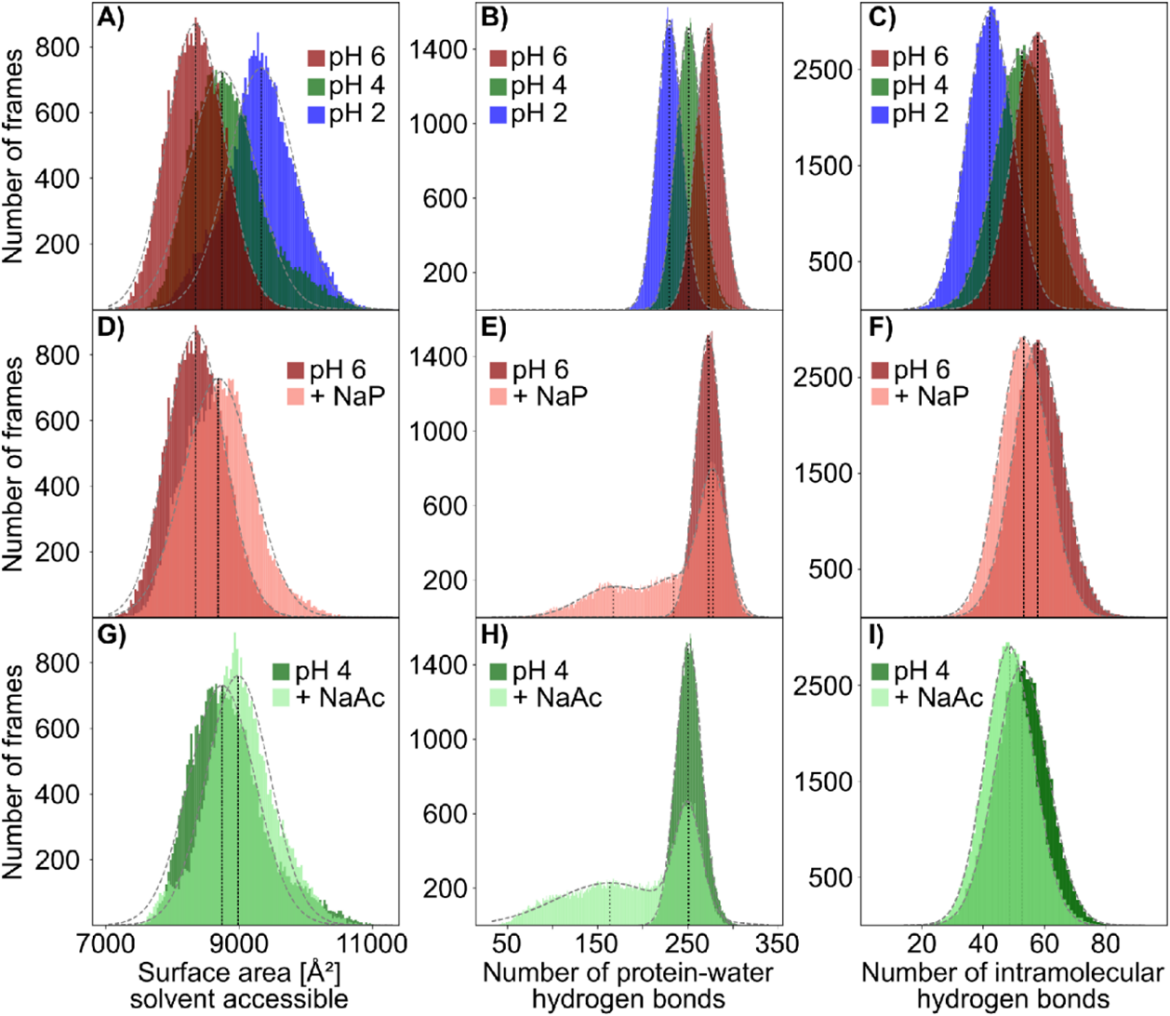
Apo-Mb solvent accessible surface area (SASA), protein–water hydrogen bonds, and intramolecular hydrogen bonds. Histograms depict the distribution of analyzed simulation frames for unbuffered simulations at pH 6 (red), pH 4 (green), and pH 2 (blue), as well as with 20 mM of either NaP buffer at pH 6 (light red) or NaAc buffer at pH 4 (light green). Gray dotted lines indicate either a Gaussian, bimodal, or trimodal fit to the data sets, with black dotted lines highlighting the peak of each fitted curve. At pH 6, 4, and 2 (A, B, C) in unbuffered solution, a decrease in the system pH causes an increase in SASA, and a reduction in the number of hydrogen bonds both with water and within the molecule, indicating a denaturation process for the protein. Upon addition of 20 mM NaP buffer (D,E,F), the protein SASA increases, as fewer intramolecular hydrogen bonds are formed compared to the unbuffered solution. Interestingly, the main peak for protein–water hydrogen bonds shows a slight shift toward more hydrogen bonds when compared to the unbuffered simulations, with two secondary peaks of frames with significantly reduced protein–water hydrogen bonds, not present in the unbuffered solution. NaAc, on the other hand (G, H and I), while also showing an increase in SASA and fewer intramolecular hydrogen bonds, has no significant increment in the main peak for protein–water hydrogen bonds, but shows more frames with low protein–water hydrogen bonds in the secondary peak.

#### Influence of Acetate Buffer at pH 4

The MG states of Apo-Mb at pH 4 have been studied extensively.^44,49,50^ The MG state is in equilibrium between two forms, *I*_a_ and *I*_b_, and is positively charged due to the protonation of the amino acid residues.^41^ Compared to the state at pH 6, the protein exhibits increased sensitivity to environmental changes due to partial unfolding. CD measurements at pH 4 showed that the NaAc buffer promotes further unfolding of Apo-Mb, resulting in lower α-helical content compared to unbuffered acidic conditions. Because NaAc is near the center of the Hofmeister series (see Figure S5) and is significantly less kosmotropic than sodium phosphate (NaP) buffer, it is expected to interact more strongly with the highly positively charged, partially folded MG state of Apo-Mb. Indeed, the MD simulations with NaAc show strong interactions with the protein surface, particularly when compared with NaP (Figure 6, see below). The thermodiffusion data of Apo-Mb in the presence of NaAc buffer are now discussed. The temperature dependence of *S*_T_ of Apo-Mb flattens and the *S*_T_ values increase with increasing NaAc concentration (see Figure 8). The thermal diffusion coefficient *D*_T_ (see Figure S3) also increases with increasing NaAc concentration. A similar trend was observed for *S*_T_ and *D*_T_ of dextran in water with increasing urea concentration.^21^ Despite the differences in these systems, in both cases the addition of a hydrophilic compound leads to a weaker temperature dependence of *S*_T_, which is typically associated with a disruption of hydrogen bonds between the solute and water. We also observed a slight decrease in the diffusion coefficient of Apo-Mb with increasing NaAc concentration (see Figure S3). The partial unfolding of Apo-Mb enhances its likelihood to aggregate, a process that might further be intensified in NaAc buffer due to charge screening.^85^ Thus, the observed decrease in *D* likely reflects partial unfolding of the protein and possible contributions from aggregation (see Section S5.4).

**Figure 8:**
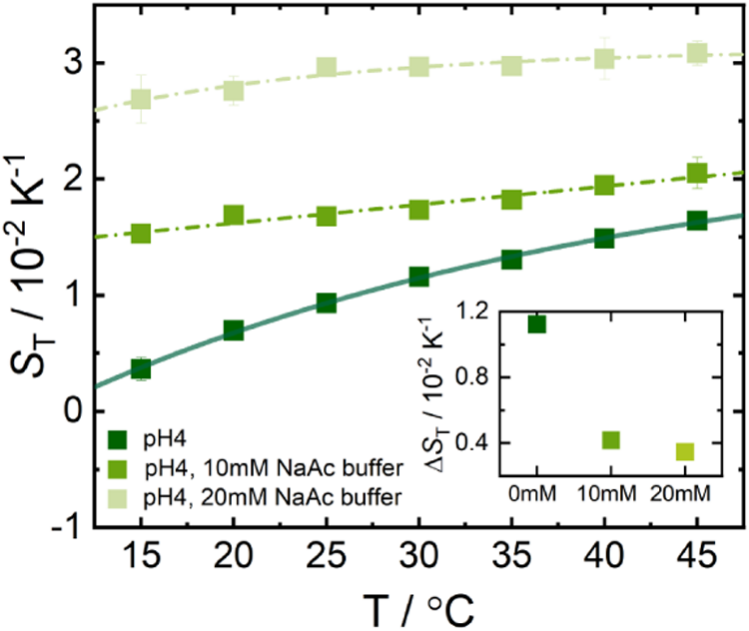
Temperature dependence of *S*_T_ of Apo-Mb with and without NaAc buffer at pH 4 (see Table 1). The dark green squares represent the ApoMb solution without buffer (pH adjusted with hydrochloric acid), while the middle and light green pentagons correspond to ApoMb in 10 mM and 20 mM NaAc buffer, respectively. The curves represent fits to eq 2, which describes the temperature dependence of *S*_T_. The inset shows the temperature sensitivity, Δ*S*_T_(Δ*T*), as a function of increasing NaAc concentration. *S*_T_(Δ*T*) is defined in Figure 4.

As indicated above, the 20 mM NaAc Apo-Mb pH 4 CpHMD simulations show strong interactions with the buffer components over all protein residues, in stark contrast to the simulations at NaP pH 6 (Figure 6). Apo-Mb has a considerably higher positive charge at pH 4, which favors further interactions with the negatively charged acetate ions (See Figure S8).

Although these multiple interaction positions between NaAc and the protein can, as in the case of NaP, explain changes in *D*, the additional interaction sites agree with the notion of a molecular shielding for charged residues, which does not seem to be the case for NaP. Similar to NaP, the inclusion of NaAc at pH 4 increased the SASA compared to the unbuffered solution, but the resulting SASA is higher than that for NaP, which could be a result of the additional reduction in α-helical structure shown above. There is a reduction in the number of hydrogen bonds with respect to the unbuffered solution as well, suggesting a decrease in direct interactions with the solvent, with a further reduction in the intramolecular hydrogen bonds than in NaP-buffered or pH 4 unbuffered solution (Figure 7).

While at first glance this distribution is similar to that with NaP, it is important to note that the main distribution peak is not shifted toward more hydrogen bonds than in the unbuffered solution, in contrast to the increase observed with NaP. Overall, NaAc increases SASA more than NaP, reduces the number of hydrogen bonds, and simultaneously influences the secondary structure of apo-Mb (Figure 2). The overall NaAc effect with the initial drop in the α-helical content caused by the interactions with the buffer could explain the higher initial *S*_T_ and the consequent drop in Δ*S*_T_ observed for NaAc in Figure 8. The effects of decreasing pH values and buffer addition on the SASA and hydrogen bonds are also reflected in shifts in the number of water molecules in the first and second water shells around the protein, which both increase upon reduction of pH and upon buffer addition (Figure S15).

The inset in Figure 8 shows that Δ*S*_T_(Δ*T*) decreases with increasing NaAc concentration at pH 4. The decrease in Δ*S*_T_(Δ*T*) is similar for 10 mM and 20 mM NaAc buffers due to their low ionic strength. As evidenced in the MD simulations, NaAc interacts strongly with the protein surface, reducing its effective charge and disrupting intra- and intermolecular hydrogen bonding compared to the unbuffered acidic condition (as evidenced through lower α-helical content). Ultimately, this facilitates the unfolding and exposure of the hydrophobic regions of the protein to the aqueous environment, reducing its overall hydrophilicity. This decrease in hydrophilicity is reflected in decreased Δ*S*_T_(Δ*T*) values and follows the observed trend: the lower the hydrophilicity of the protein, the lower is Δ*S*_T_(Δ*T*).

A similar behavior has also been observed for streptavidin–biotin as Δ*S*_T_ of the complex was reduced compared to the isolated streptavidin.^22^ The streptavidin–biotin complex was less flexible compared to free streptavidin, so that the conformational entropy of the complex was substantially reduced, while an increase in the entropy of the hydration layer was observed.^22^ Liese et al.^108^ observed a similar phenomenon in stretched (rigid) versus flexible poly­(ethylene glycol) (PEG) chains, where entropic hydration effects nearly compensated for chain conformational entropy. Specifically, water molecules formed fewer hydrogen bonds in the hydration layer of the rigid, stretched PEG than in the flexible PEG coil.

#### Correlation between Circular Dichroism and IR-TDFRS

The next step was to compare the CD results with the IR-TDFRS results. Considering the change in structure posed by the changes in pH and buffer interactions, we wanted to evaluate if there is a correlation between the calculated α-helical content and the thermophilicity in terms of thermal sensitivity, which is a reliable indicator of the hydrophilicity of solute molecules in water.^5,11,23,109^ To do this, we plotted Δ*S*_T_(Δ*T*) against the α-helical content as shown in Figure 9.

**Figure 9:**
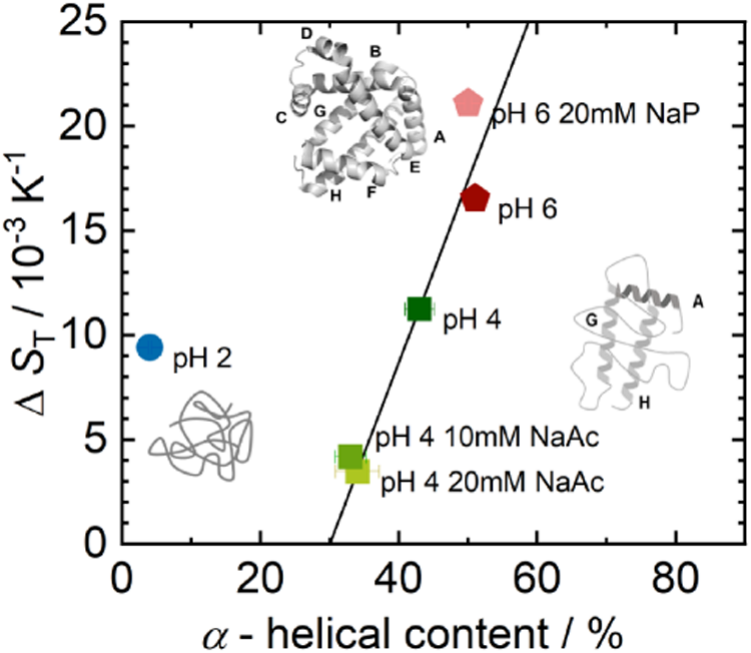
Temperature sensitivity, Δ*S*_T_(Δ*T*), plotted as a function of the α-helical content. The plot shows a strong correlation between the two parameters. Δ*S*_T_(Δ*T*) is defined in Figure 4.

We find that Δ*S*_T_(Δ*T*) correlates linearly with the α-helical content of Apo-Mb over a wide range, strongly suggesting that it reflects the folding state and hydrophilicity of Apo-Mb. This occurs because, in an α-helical conformation, hydrophobic residues are typically buried within the protein core, while hydrophilic residues are exposed to the surrounding aqueous environment. In addition, previous studies show that the α-helical content of a protein is a main determinant of protein diffusion characteristics.^40^ This correlation is also observed in our results. In particular, the temperature sensitivity of the thermodiffusive behavior, expressed by Δ*S*_T_(Δ*T*), decreases in the following order as the hydrophilicity of Apo-Mb decreases (which is achieved by lowering the pH): Δ*S*_T_ (pH 6) > Δ*S*_T_ (pH 4) > Δ*S*_T_ (pH 2).

Our MD simulations suggest that the NaP buffer, compared with the unbuffered solution, has the potential to increase the number of hydrogen bonds of the protein with water, while not affecting the α-helical content of the protein. This results in increased hydrophilicity and a higher value of Δ*S*_T_(Δ*T*).^48,51^ Conversely, NaAc buffer promoted protein solubilization, which was reflected in the reduction of α-helical content and in water hydrogen bonds. At pH 4, the reduced hydrophilicity caused by the increased exposure of the hydrophobic regions led to a significantly lower Δ*S*_T_(Δ*T*) value in the presence of NaAc buffer compared to that in unbuffered acidic solution at the same pH. At pH 2, the protein structure was largely disrupted, with a significant decrease in the α-helical content and an increase in the net positive charge. This perturbation led to a deviation from the observed trend between Δ*S*_T_(Δ*T*) and the α-helical content.

## Conclusion

TDRFS is a highly sensitive technique for probing protein hydration in solution because it is very sensitive to the nature of solute–solvent interactions. This is especially true when hydrophobic, hydrophilic, and charged species are involved in the protein structure, as well as the interaction of the protein with buffer components. In this context, our study investigates the influence of conformational changes on the thermodiffusion behavior of Apo-Mb using the IR-TDFRS technique. By adjusting the pH and changing the buffer conditions, we were able to generate differently folded states of Apo-Mb. Apo-Mb undergoes pH-dependent conformational shifts: It maintains a folded, neutral state at pH 6, transitions to a positively charged, partially folded MG state at pH 4, and becomes strongly positive and acid unfolded at pH 2. These structural changes were confirmed by CD analysis and CpHMD simulations, which revealed a progressive decrease in the α-helical content with decreasing pH (pH 6 > pH 4 > pH 2), and a reduction in the number of hydrogen bonds between the protein and water. We found that the α-helical content is strongly correlated with Δ*S*_T_(Δ*T*), which is a measure of the protein folding state and hydrophilicity.^5,27,109^ Reducing the α-helical content is considered to decrease hydrophilicity (pH 6 > pH 4 > pH 2) and to decrease the temperature sensitivity of *S*_T_ (Δ*S*_T_). (Δ*S*_T_(Δ*T*) (pH 6) > Δ*S*_T_(Δ*T*) (pH 4) > Δ*S*_T_(Δ*T*) (pH 2)).

The buffer type also plays a significant role in modulating the structural and diffusion properties of apo-Mb. At pH 6, phosphate buffer retains the α-helical content, but promotes aggregation of the protein due to electrostatic screening, which is reflected in a lower diffusion coefficient. CpHMD simulations showed how NaP tends to interact sparsely at dedicated spots with the protein, mainly close to the heme cavity, and seems to foster protein–water hydrogen bond formation. By contrast, acetate buffer at pH 4 decreases the α-helical content of the protein below the values obtained in unbuffered conditions, causing a decrease in α-helical content in helices A, E, and G. These changes cause a decrease in the temperature sensitivity of *S*_T_ and favors the unfolding of the protein in solution. Increasing the concentration of the acetate buffer at pH 4 further decreases the temperature sensitivity of *S*_T_.

Overall, we observed a strong correlation between Δ*S*_T_(Δ*T*) and α-helical content; Δ*S*_T_(Δ*T*) decreases steadily with decreasing hydrophilicity and α-helical content of Apo-Mb, showing a strong correlation with structural changes evidenced by CpHMD simulations. These results highlight the complex interplay between the structural state of Apo-Mb, pH, buffer composition, and thermodiffusion behavior and provide valuable insights into protein hydration.

## Supplementary Material



## Notes

Relation between hydrophilicity and solvent-accessible surface area of proteins including ApoMb, overlap concentration of ApoMb, electrophoresis measurements of ApoMb, refractive index contrast measurements, and temperature sensitivity of *S*_T_ at different solution conditions, diffusion coefficient, and thermal diffusion coefficients at pH 2 and pH 4, additional information on buffers, parameters describing the temperature dependence of *S*_T_. Further information is provided on the simulation step parameters, the estimated p*K*_a_ values of each titrated side chain, the average α-helical content per solvent condition, the average net charge of apo-Mb during CpHMD simulations, and the proportion of simulation time that each residue spends as part of an α-helix; convergence of the simulations with RMS average correlation (RAC) plots, the convergence of the average α-helical content per simulation condition, the discovery of conformational clusters over time for both the full 3 μs simulations and the last 500 ns, and the changes in the number of water molecules in the 1st and 2nd hydration shells (PDF)
