# Supplementary material for: Influence of α‑Helical Content on the Thermodiffusion of Apomyoglobin
Source: Langmuir. 2025 Oct 15;41(42):28322–34. doi: 10.1021/acs.langmuir.5c02797 (PMC12573798; doi:10.1021/acs.langmuir.5c02797)
Supplement: Supplementary file 1 [file la5c02797_si_001.pdf]

Supporting Information: Influence of α-Helical Content on the Thermodiffusion of Apomyoglobin

Binny A. Rudani, Steffen Docter, Stephan Schott-Verdugo, Johan Buitenhuis, Andreas M. Stadler, Holger Gohlke and Simone Wiegand

Contents

S1 Overlap concentration S3

S2 Electrophoretic charge on Apo-Mb S3

S3 Refractive index increments S4

S4 Diffusion coefficient and thermal diffusion coefficient S5

S5 General information on buffers S7

S5.1 Choice of the buffers for the CD measurement S7
S5.2 Influence of the buffer components on the TDFRS signal of Apo-Mb S7
S5.3 $\log P$ values of buffers S9
S5.4 Hofmeister effect of buffers on protein S9

S6 Temperature sensitivity of S_{T} S11

S7 Average α-helical content during CpHMD simulations S13

S8 Average net charge of Apo-Mb calculated from CpHMD simulations. S15

S9 Average α-helical content per residue S16

S10 Simulation setup parameters. S19

S11 Predicted pKa values across simulation conditions. S20

S12 RMS average correlation analysis S22

S13 Cluster discovery analysis over the last 500 ns of each simulation S23

S14 Cluster discovery analysis over the full simulation time **S25**

S15 Convergence of the average α-helical content per simulation condition **S27**

S16 Distribution of water molecules in the 1st and 2nd water shells **S28**

S1 Overlap concentration

The radius of gyration of Apo-Mb is about $R_g = 2$ nm, while in the denatured state it can reach $R_g = 3.5$ nm. [1]. The overlap concentration $c^* = M/(4/3 N_A \pi R_g^3)$ is the boundary between the dilute and semi-dilute range of a polymer solution [2]. We have therefore calculated $c^* \approx 840$ g/L ($\sim 50\,mM$) and $c^* \approx 160$ g/L ($\sim 9\,mM$), which in both cases is far above our highest concentration of 7 g/L ($\sim 0.4\,mM$), so that we are clearly in the dilute range.

S2 Electrophoretic charge on Apo-Mb

The ζ-potential measures the surface charge of particles in suspension and serves as a key indicator of colloidal stability in materials science. A high ζ-potential promotes electrostatic repulsion between particles, thereby reducing aggregation and enhancing long-term dispersion stability. However, proteins differ fundamentally from ideal colloidal particles. Unlike uniform, spherical colloids, proteins often have irregular, asymmetrical structures with uneven surface charge distribution and side chains protruding from the core. These structural complexities influence how electrostatic forces affect stability.

The ζ-potential of aqueous Apo-Mb was measured at pH 2, 4, and 6, corresponding to different folded states. An aqueous solution of 4.5 mg/mL ($\sim$ 0.24 mM) Apo-Mb was measured using a Malvern Zetasizer 2000 with a dip cell at 25°C. Measurements were performed in triplicate and the mean values were used for data analysis.

The ζ-potential showed an increase with decreasing pH, reflecting a more positively charged surface at lower pH values. Specifically, the ζ potential values were 26.3±1.5 mV in the folded state (pH 6), 44.7 mV±1.3 in the partially folded MG state (pH 4), and 51.4±4 mV in the acid unfolded state (pH 2). The corresponding electrophoretic charge Z on Apo-Mb in terms of elementary unit charge was calculated from the ζ values using the following equation [3]:

$$Z = (4\pi\epsilon_0\epsilon_r a\,(1+\kappa a)\,\zeta)/e \tag{S1}$$

where ϵ_0 is the permittivity in vacuum, ϵ_r is the relative permittivity of the medium, a is the radius of Apo-Mb, e is the elementary charge, and κ^{-1} is the Debye-Hückel screening length, which is a measure of the thickness of the electrical double layer surrounding a particle in a colloidal system. The calculated values of charge were +2.8 at near native folded conditions (pH 6), +4.6 in the partially folded MG state (pH 4), +8 in the acid unfolded state (pH 2). Also, in line with the whole protein charge determined in the constant pH MD simulations. The same trend is also found in the literature, where the values were determined by mass spectrometry or simulations [4, 5, 6, 7]. The absolute values do not agree, which may be related to the fact that these techniques work in the gas phase, while our measurements were performed in an aqueous solution.

At pH 2, proteins exhibit a higher net positive charge, resulting in an increased ζ-potential and enhanced interparticle repulsion, which reduces aggregation and favors dispersion. Nonetheless, the high protonation of side chains at this low pH can induce intramolecular electrostatic repulsion, destabilizing the native conformation of the protein. In contrast, at pH 6, the reduced protonation leads to a lower surface charge and ζ-potential, but it also alleviates internal electrostatic stress, thereby preserving the native fold and structural integrity of the protein. Thus, while electrostatic repulsion at pH 2 favors colloidal stability by minimizing aggregation, the preservation of functional,

Table S1: Charge on Apo-Mb.

**Apo-Mb**	**Charge** this work	**Charge** Reference	**Technique used**	**Average net Charge** simulation (see Section S8)
at pH2	+8	+19	ESI-MS[8],	$+27.0 \pm 0.8$
at pH4	+4.6	+10 - +14	MWC and LL model, ESI-MS[6, 8]	$+16.7 \pm 1$
at pH6	+2.8	+9	ESI-MS[8],	$+4.8 \pm 0.8$

native structure is more favorable at pH 6. This balance reflects the unique physicochemical properties of proteins, where both colloidal behavior and conformational stability must be considered in assessing overall stability.

S3 Refractive index increments

Refractive index contrast factors are required to calculate S_{T}. The refractive index as a function of concentration was measured using an Abbe refractometer (Anton Paar Abbemat MW) at a wavelength of 632.8 nm. For all Apo-Mb solutions, refractive indices were measured at five concentrations around the desired concentration. The slope of the linear interpolation of the refractive indices as a function of concentration gives $(\partial n/\partial c)_{p,T}$. Figure S1 shows the decrease of the refractive index increment $(\partial n/\partial c)_{p,T}$ with temperature for aqueous Apo-Mb solutions.

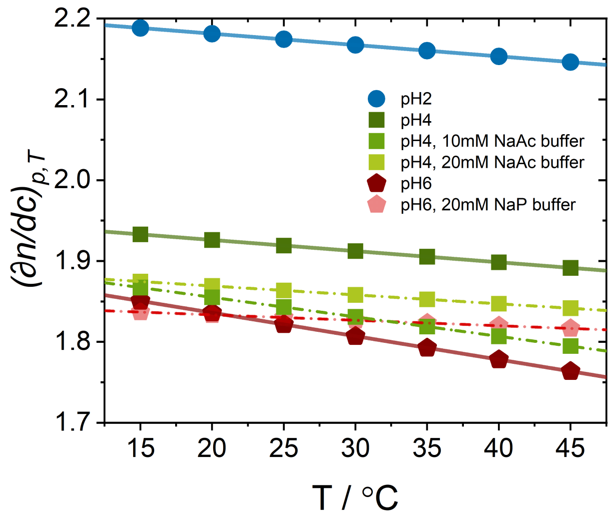

Figure S1: Temperature dependence of $(\partial n/\partial c)_{p,T}$ at 9 mM Apo-Mb solution. The lines are linear fits.

The change of refractive index with temperature $(\partial n/\partial T)_{p,c}$ was measured interferometrically [9]. The measurements were performed in a temperature range of 15-45°C with a heating rate of 1.06 mK/sec. The refractive index varied linearly with temperature in the range studied. Figure S2 shows the decrease in refractive index $(\partial n/\partial T))_{p,c}$ with temperature for aqueous Apo-Mb solutions.

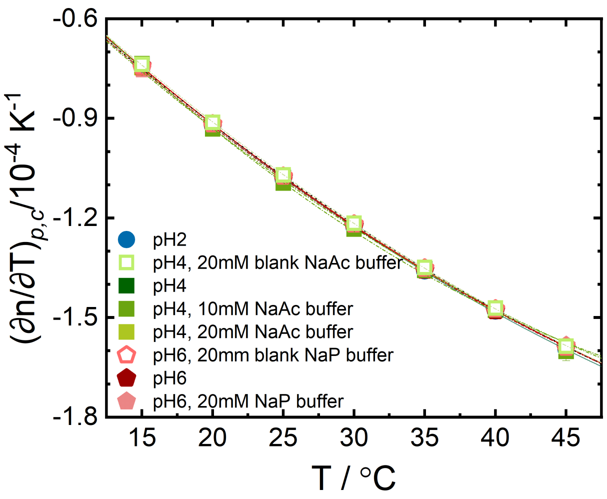

Figure S2: Concentration dependence of $(\partial n/\partial T))_{p,c}$ for aqueous Apo-Mb solutions over the temperature range from 15°C to 45°C. The lines are a second order polynomial fit.

S4 Diffusion coefficient and thermal diffusion coefficient

Figure S3 shows the temperature dependence of D_{T} and D in unbuffered pH2 and pH4 solutions and in the presence of 10mM and 20mM NaAc buffer at pH4. For all solution conditions, D_{T} and D increase with increasing temperature. This trend indicates that Apo-Mb diffuses faster with increasing temperature, probably due to the lower viscosity of the solution at higher temperatures [10, 11]. Furthermore, for unbuffered solution, the value of D decreases with increasing α-helical content in the order: (pH2 > pH4 > pH6).

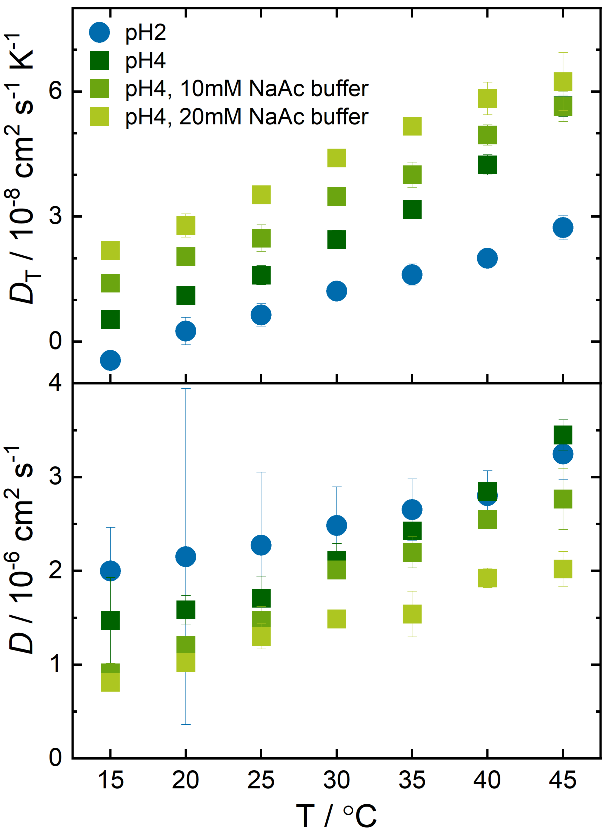

Figure S3: Temperature dependence of D_{T} and D, measured for 9 mM Apo-Mb with and without buffer. The blue circle and the dark green square represent the Apo-Mb solution at pH 2 and pH 4 without buffer (adjusted with hydrochloric acid). The medium and the light green square correspond to Apo-Mb in 10 mM and 20 mM NaAc buffer at pH 4, respectively.

S5 General information on buffers

S5.1 Choice of the buffers for the CD measurement

Buffers were selected within specific pH ranges to ensure maximum buffering capacity, based on their respective pKa values (see Table S2). At pH 6 and pH4, NaP and NaAc were the preferred buffer respectively due to its strong biological relevance. For measurements at pH 4, acetate buffer was selected because because of their low UV absorbance, which permits spectral analysis in the 180–260 nm range. To evaluate potential effects specific to the buffer, citrate buffer at pH 4 was also tested. However, in the case of apomyoglobin, citrate buffer hindered protein solubilization and promoted aggregation, in contrast to acetate buffer. This observation is supported by previous studies reporting that citrate buffers can induce biomolecular aggregation [12, 13]. Furthermore, the high voltage and absorption at lower wavelengths limited the analysis range of the CD spectrum to 188–260 nm. This resulted in large uncertainty in the α-helical content, preventing us from using the citrate buffer. Thus, acetate buffer is the most appropriate choice for CD measurements at pH 4.

Table S2: Overview of the used buffers

buffer	acid-base pair	pKa	buffering range
NaAc	CH_3COOH/CH_3COO^-	4.76	3.76-5.76
NaP	$H_3PO_4/H_2PO_4^-$	2.15	1.15-3.15
NaP	$H_2PO_4^-/HPO_4^{2-}$	7.20	6.2-8.2
NaP	HPO_4^{2-}/PO_4^{3-}	12.35	11.35-13.35

S5.2 Influence of the buffer components on the TDFRS signal of Apo-Mb

The Soret coefficient of Apo-Mb was determined by treating the systems as a pseudobinary system and neglecting the contribution of the buffer. This is justified only if the buffer does not contribute to the concentration signal. Therefore, we measured the buffer at the same concentration as in the protein solution, but without the protein. Figure S4 shows the raw TDFRS data and their respective residual plots for the 20mM NaAc (A) and 20mM NaP (B) buffers at 25°C. The solid line through the data points is a fit according to Eq. 2 in the main manuscript. The green and red points in the residual plots are within 2σ and show no systematic deviations. The amplitude of the concentration signal was flat for both buffers, indicating that the buffers have no discernible effect on the TDFRS measurements of Apo-Mb.

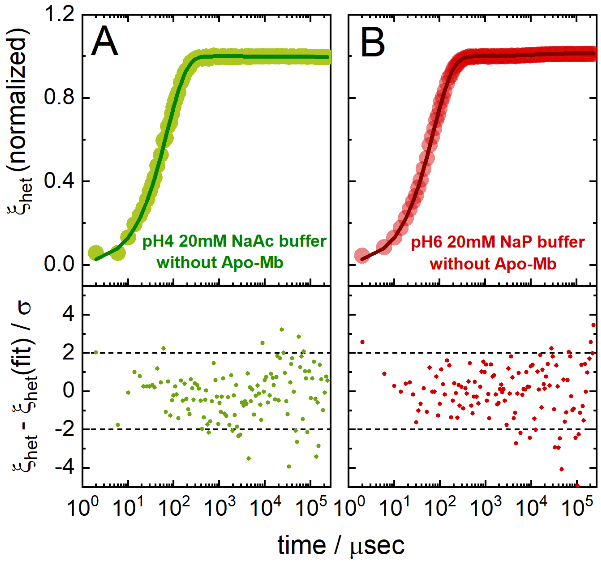

Figure S4: Normalized heterodyne diffraction intensities of (A) 20mM NaAc and (B) NaP buffer at 25°C. Filled circles represent the data points, and the solid dark line indicates the fit according to Eq. 2. The residuals are shown as dots in the lower plots.

S5.3 $\log P$ values of buffers

$\log P$ values were calculated using Calculator Plugins, Marvin 24.1.2, ChemAxon (http://www.chemaxon.com), based on the method of Vishwanadhan *et al.* [14]. For the computation of $\log P$ respectively $\log D$ for charged compounds taking into account the distribution of components at a particular pH: i) electrolyte concentration (Na^+, K^+, and Cl^-) is set to be zero mol/dm^3, ii) $\log P$ ($\log D$) value of individual salts used for buffer preparation is summation of cations and anions ionic at the corresponding pH.

The majority component of the phosphate buffer, sodium dihydrogen phosphate, has a lower $\log P$ value than the majority component of the acetate buffer, acetic acid. This shows that the phosphate buffer is more hydrophilic than the acetate buffer. Further details for $\log P$ calculations are listed in Table S3, which shows also the average values according to the mole fractions x_i of the buffer components.

$$\log P_{\text{average}} = x_1(\text{NaH}_2\text{PO}_4) \cdot \log P(\text{NaH}_2\text{PO}_4) + x_2(\text{Na}_2\text{HPO}_4) \cdot \log P(\text{Na}_2\text{HPO}_4) \tag{S2}$$

Table S3: Log P values of buffer.

**Salts**	**mole fraction**	$\log P$	$\log P_{\text{average}}$
NaH_2PO_4	0.86	-4.7	-4.8
Na_2HPO_4	0.14	-5.47	
CH_3COONa	0.23	-1.05	-0.45
CH_3COOH	0.77	-0.28	

S5.4 Hofmeister effect of buffers on protein

Phosphate ions such as $H_2PO_4^-$ and HPO_4^{2-} with their high hydration enthalpies (-522 kJ/mol and -1170 kJ/mol, respectively) exhibit pronounced hydrophilicity and strong interactions with water molecules, leading to extended solvation shells [15]. This pronounced hydrophilicity also increases the surface tension of water in the presence of these anions, which reduces the tendency of water to penetrate hydrophobic regions inside the proteins and thus contributes to the stability of the proteins or their precipitation.

In contrast, the acetate ion with a less negative hydration enthalpy of about -425 kJ/mol interacts weaker with water due to its lower charge density and smaller size compared to phosphate ions [16]. In addition, the presence of a hydrophobic methyl group gives the acetate a mixed hydrophilic-hydrophobic character [17]. Hydrophobic anions such as acetate tend to interact preferentially with the hydrophobic regions of proteins, disrupting the surrounding water structure and thereby may destabilize proteins to some extent. This depends on the type of protein. This contrasting behavior highlights the importance of buffer ion-water interactions for protein stability and solvation dynamics[18, 19].

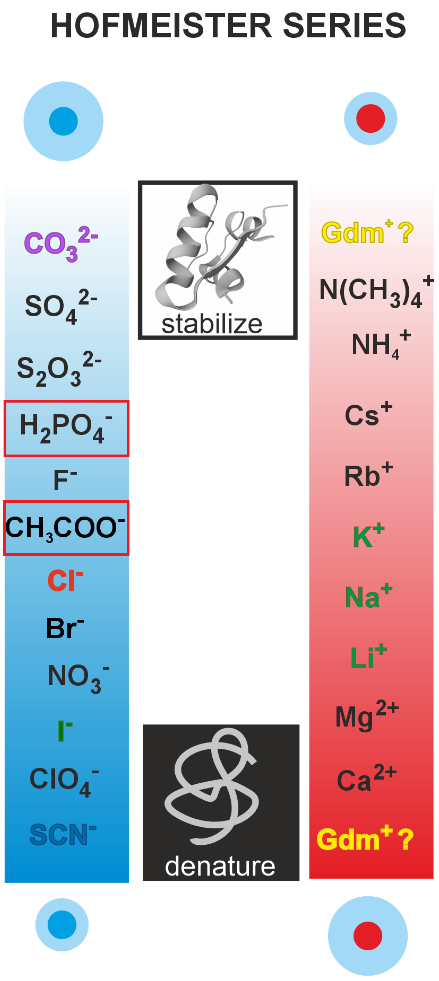

Figure S5: The Hofmeister series shown here arranges the anions (left panel) and cations (right panel) in the order of protein stabilization. The red rectangular box indicates the buffer anions. The ions at the bottom promote protein denaturation, while the ions at the top stabilize the protein structure. We have marked the more hydrophilic ions with a thicker light blue ring, while the thinner ring indicates less hydrophilicity. It is important to note that the hydrophilicity of anions and cations is inversely proportional to their effect on protein denaturation. Further explanations can be found in the text above.

S6 Temperature sensitivity of S_T

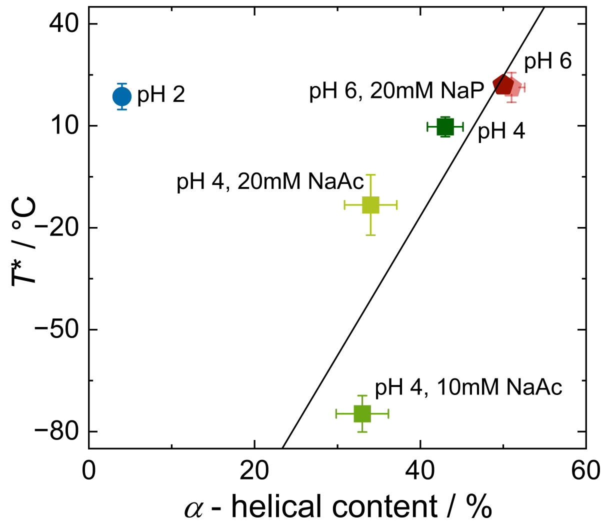

Figure S6: Inversion temperature T^*, at which the S_T changes sign, is plotted as a function of the α-helical content. The plot shows a strong correlation between the two parameters.

The typical uncertainty of the fitted parameters for S_T^∞ is 10%, the amplitude A is about 5%, while for T_0 it is about 5% (see Table S4). These parameters, derived from the empirical expression (Eq. 3), are quite consistent for the unbuffered solution. The values for S_T^∞, $-A$ and T_0 decrease with decreasing pH. However, the trend shows deviations in the presence of buffer. Under the condition of 20 mM NaAc buffer, the uncertainty is significantly larger (T_0: 51% and A: 40%) compared to the other solution conditions. In addition, the data at pH 6 in the presence of NaP buffer could not be determined at 45°C due to protein aggregation. For a more consistent comparison over a given temperature range and to minimize the large uncertainties associated with the fitted parameters in the presence of buffer, it is often more reliable to describe the temperature sensitivity of S_T by $\Delta S_\mathrm{T}(\Delta T)$= S_T(40°C) - S_T(15°C). This approach provides a clearer representation of the temperature dependence, especially when buffer conditions cause fluctuations.

Furthermore, T^* denotes the temperature at which S_T changes sign, indicating a transition from thermophilic to thermophobic or thermophobic to thermophilic behavior with increasing temperature [20]. Here T^* increases with an increase in α-helical content (see Fig. S6), following the trend: T^* (pH 6, NaP buffer) $\approx$ T^* (pH 6) $>$ T^* (pH 4) $>$ T^* (pH 4, 20mM NaAc)$>$ T^* (pH 4, 10mM NaAc). At pH 2, the protein structure was largely disrupted, leading to a deviation. The correlation between the α-helical content and the sign-change temperature T^* is uncertain, because T^* can only be determined by extrapolation. The largest deviation occurs in the measurement at pH 4 with 10 mM NaAc, where linear extrapolation yields a very low T^*. We expect the same physics to be present in the temperature sensitivity of the Soret coefficient $\Delta S_\mathrm{T}(\Delta T)$ and T^*. However the correlation with $\Delta S_\mathrm{T}(\Delta T)$, which is always accessible with the experimental range, is a more robust property to consider.

Table S4: Fitting parameters obtained for Apo-Mb by means of Eq. 3 in the main manuscript under different conditions of the solution. Additionally, we calculated the sign change termperature T^*.

protein	S_T^∞ / 10^{-2} K^{-1}	$-A/10^{-2}$	$T_0/°C$	$T^*/°C$
Apo-Mb at pH2	1.18 ± 0.12	2.75 ± 0.12	21.90 ± 3.42	18.6 ± 3.8
Apo-Mb at pH4	2.41 ± 0.19	3.28 ± 0.08	31.43 ± 4.49	9.7 ± 2.9
Apo-Mb at pH4, 10mM NaAc buffer*	-	-	-	-74.75 ± 5.34
Apo-Mb at pH4, 20mM NaAc buffer	3.12 ± 0.10	1.25 ± 0.49	14.51 ± 7.42	-13.3 ± 8.9
Apo-Mb at pH6	2.95 ± 0.27	5.18 ± 0.16	37.82 ± 4.23	21.3 ± 4.36
Apo-Mb at pH6, 20mM NaP buffer	2.7 ± 0.15	6.10 ± 0.06	26.91 ± 1.73	22.0 ± 2.1
0.5M NaP buffer (without ApoMb)	0.81 ± 0.049	0.67 ± 0.02	27.54 ± 5.53	-5.2 ± 2.14
0.5M NaAc buffer (without ApoMb)*	-	-	-	-

* Fitting the curve of Apo-Mb at pH4, 10mM NaAc buffer and 0.5M NaAc buffer (without Apo-Mb) using Eq. 3. was unsuccessful as the curve is relatively flat.

S7 Average α-helical content during CpHMD simulations

For all five simulated conditions (unbuffered pH 6, 4, 2, as well as 20 mM NaP buffer at pH 6 and 20 mM NaAc buffer at pH 4), Figure S7 shows the secondary structure for every protein residue, determined in 200 ps steps using the DSSP implementation of CPPTRAJ [21, 22]. The fraction of residues classified as α-helical by DSSP was calculated for eleven replica over the 3 μs long CpHMD simulations and averaged for every condition. The plotted timeseries of α-helical fractions shows a clear decrease for all conditions from the initial starting structure of horse-heart Holo-Mb (PDB-ID: 2V1K) with all non-protein components removed. At pH 6, the averaged α-helical content of both the unbuffered and 20 mM NaP buffer conditions reaches the targeted value of 51% $\pm$ 1.6% and 50% $\pm$ 0.5%, respectively, after roughly 1 μs (see Table 1 in the main manuscript). The unbuffered simulations at pH 4 reach an average α-helical content of 43% $\pm$ 2.1% after 1.6 μs, while the simulations at pH 4 with 20 mM NaAc buffer only reach the upper limit of the experimentally determined range of 34% $\pm$ 3.2% after 2.2 μs. The simulations at pH 2 do not reach the targeted α-helical content of 4% $\pm$ 0.3% within the limits of the simulation time. While the data distribution in the simulations in both pH 6 conditions becomes reasonably small from around 2.3 μs on, all other simulated conditions show considerable deviations between each replica for the full simulation time. This is expected behavior, since Apo-Mb is known to adopt a structurally flexible molten globule state at pH 4. The unfolding at pH 2 takes place at different speeds in the independent simulation replica, leading to a similarly large data distribution.

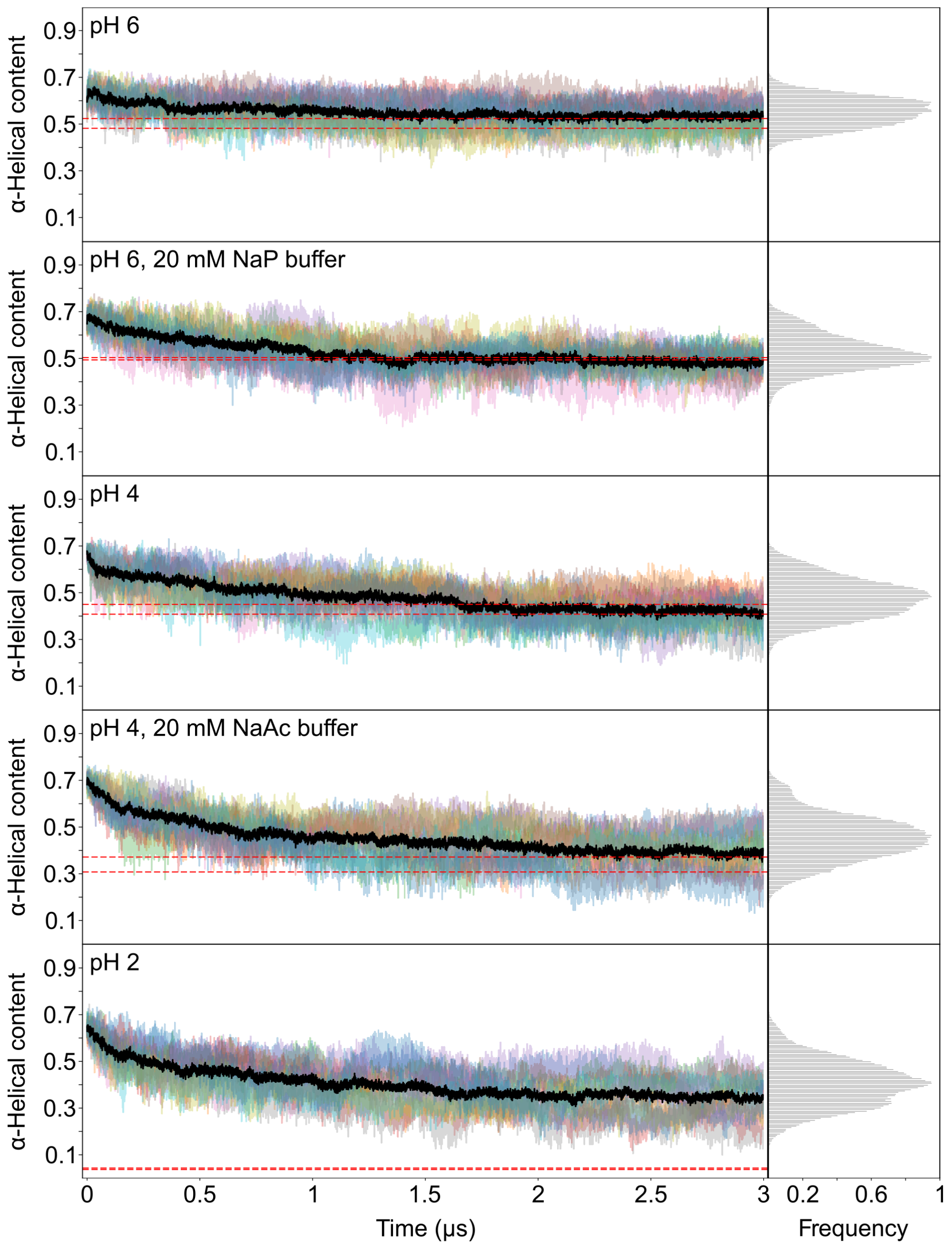

Figure S7: Relative α-helical content per simulated condition. Each timeseries depicts the fraction of α-helical secondary structure elements of Apo-Mb over the simulated solvent conditions at pH6, 4 and 2 (unbuffered), as well as with 20 mM NaP buffer at pH 6 and 20 mM NaAc buffer at pH4. For each condition, 11 independent simulation replica of 3 μs length were performed (coloured lines). The average α-helical content is depicted in black. The upper and lower range of the α-helical content measured for each condition via CD-spectroscopy is indicated by red dotted lines. For each timeseries, the data distribution over all replicas is depicted to the right of the plot as histograms.

S8 Average net charge of Apo-Mb calculated from CpHMD simulations.

Figure S8 shows the averaged protein net charge for the last 500 ns of the CpHMd simulations for the five simulated solvent conditions. We used cphstats from AmberTools24 [23] to determine the protonation states over time for the titratable residues aspartate, glutamate, histidine, lysine and tyrosine. Due to their high pka values, asparagine residues were considered as positively charged and prolines as neutral. Cysteines as potentially titratable residues do not occur in the Apo-Mb sequence. For each residue i, we sum over all possible charge states $q_{i,j}$, multiplied by their fraction of occurrence $f_{i,j}$. By summing over all charged residues, we obtain the total net charge Q of the protein for each condition:

$$Q = \sum_{i=1}^{N} \sum_{j} f_{i,j} \cdot q_{i,j} \tag{S3}$$

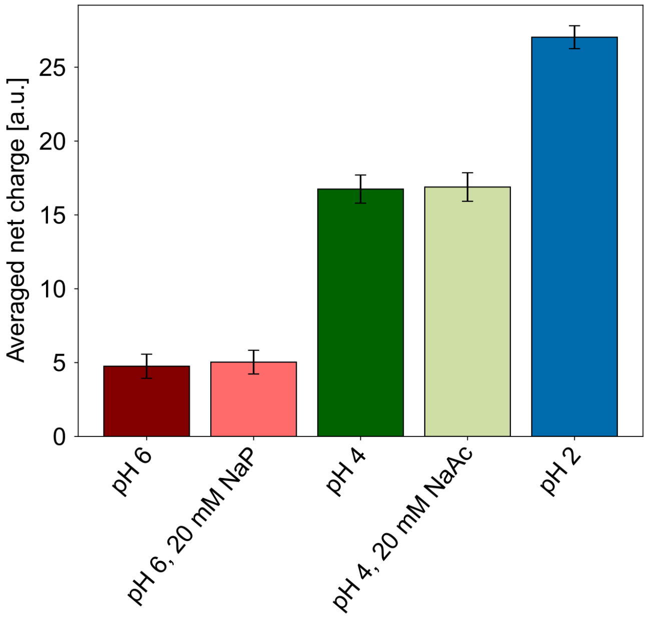

Figure S8: Net charge of Apo-Mb over the last 500 ns simulation time for unbuffered solvent at pH 6 (red), pH 4 (green) and pH 2 (blue), as well as with 20 mM NaP buffer at pH 6 (light red) and 20 mM NaAc buffer at pH 4 (light green).

We find a clear increase in net charge with decreasing pH, with calculated average values for the unbuffered contions of +4.8 ± 0.8 at pH 6, +16.7 ± 1 at pH 4 and +27.0 ±0.8 at pH 2, in line with the general trend in Table S1 , but slightly lower at pH 6 and higher at pH 2 than the net charges determined by Konermann *et al.* [8] . We also see no significant changes in net charge introduced by addition of either 20 mM NaP buffer at pH 6 (+5.0 ± 0.8) or 20 mM NaAc buffer at pH 4 (+16.9 ± 1). This is interesting, as while at pH 6, buffered and unbuffered Apo-Mb is thought to retain its fold, at pH 4 the addition of 20 mM NaAc buffer leads to stronger unfolding compared to the unbuffered condition. While our simulations reflect this increased unfolding as a decrease in

α-helical content (Figure S7), this does not seem to translate to conformational changes affecting the net charge.

S9 Average α-helical content per residue

Using the DSSP implementation in CPPTRAJ, we calculated the fraction of simulation time each residue spends as part of an α-helix for the last 500 ns seconds of each simulation replica. For all five simulated solvent conditions (unbuffered at pH 6,4,2 and with 20 mM of NaP or NaAc buffer at pH 6 and 4, respectively), the per residue α-helical fraction was averaged over eleven replicas. Figure S9 compares the per residue α-helical content at each simulated pH value. At pH 6, Apo-Mb retains the overall fold of Holo-Mb for all helices except C and F, as known from the work of Picotti *et al.* [24]. Between buffered and unbuffered simulations, few notable differences become apparent, with parts of helix F retaining the helical conformation for slightly larger fractions of the simulation when NaP is present, while the first half of helix B seems to be more stable in unbuffered simulations. Interestingly, we see a very similar stabilization of helix F upon addition of NaAc buffer at pH 4 when compared to pH 6, coupled with a slight destabilization of helix A by the acetate ions. Overall, Helices B, D and parts of helix G are clearly destabilized at pH 4, when compared to pH 6 simulations. Finally, while the 3 μs simulation time at pH 2 does not allow full unfolding of the protein, we see further destabilization of helices A-E and G, compared to pH 4. Helix H stays remarkably stable over the simulation time, while helix F even is more stable than in all other simulated systems. As the proteins is not fully unfolded, ascribing a deeper meaning to the findings at pH 2 from our CpHMD simulations is not sensible without extending simulation times beyond reasonable computational costs. We further highlight residue specific effects of the added buffers by evaluation the per residue difference $D(i)$ between the averaged α-helical fraction unbuffered $H_{ub}(i)$ and with 20 mM of NaP or NaAc $H_b(i)$.

$$D(i) = H_{ub}(i) - H_b(i) \tag{S4}$$

In both comparisons, we find mostly statistically non-significant differences with a p-value cut-off of 0.001 in Figure S10. At pH 6, the addition of NaP buffer significantly increases the α-helical fraction of leucine 86 and glutamine 91. Both residues belong to helix F of the protein. Analysis of protein-buffer contacts over the simulations (see Figure 6 in the main text) shows no common direct contact between NaP and these two residues, however, neighboring residues 98 and 96 have the highest averaged amount of buffer contacts (9% $\pm$ 3% and 8% $\pm$ 3%, respectively) with NaP out of all residues, while lysine 87 also shows buffer contacts for 5% $\pm$ 2% of the simulations. Since these residues either belong to helix F (residues 82-96) or are directly next to it, a stabilizing effect of NaP buffer on helix F can be hypothesized. Opposed to this, the addition of NaAc buffer at pH 4 induces a significant decrease in helical content for lysine 77 at the end of helix E (residues 58-77). This does coincide with high buffer interactions with isoleucine 75 (13% $\pm$ 2%) and leucine 76 (13% $\pm$ 2%) in Figure 6 in the main text. A direct interaction of the positively charged side chain of lysine 77 with the negatively charged acetate ions occurs in the simulations (8% $\pm$ 8%) but cannot be confirmed from the contact analysis alone due to the high standard deviations stemming from the incomplete unfolding of individual simulation replicas at pH 4 with 20 mM NaAc buffer (see S7).

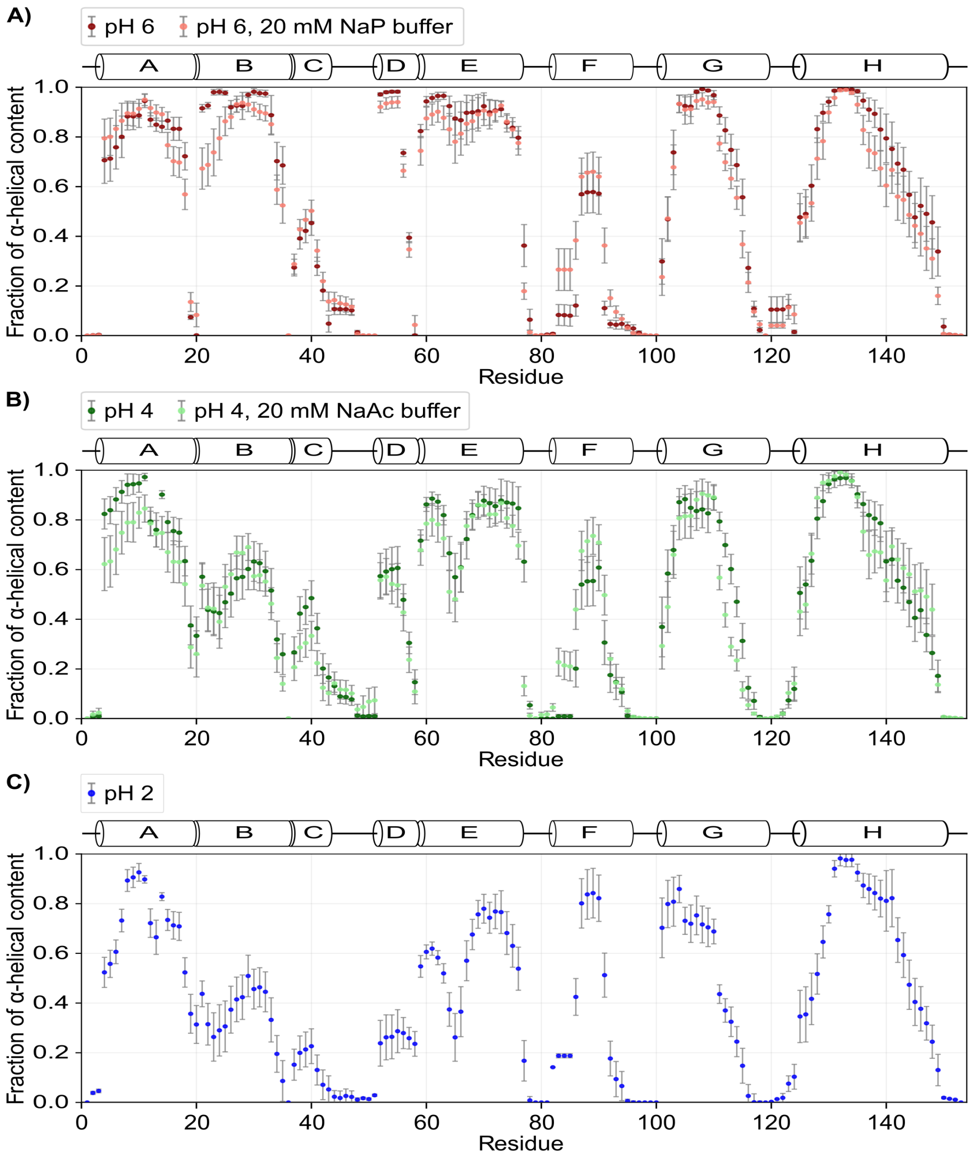

Figure S9: Per residue averaged α-helical fraction of Apo-Mb. For every protein residue, the α-helical content was calculated using DSSP and averaged over the last 500 ns simulation time of 11 replica with the standard error of the mean indicated as error bars. Helix names and positions of the X-ray crystal structure of holo-myoglobin (PDB-ID: 2V1K) are schematically referenced above each graph. A) Simulations at pH 6 in unbuffered solution (red) or with 20 mM NaP buffer (light red) overall show high similarities in α-helical content, with the most notable differences at the helix termini. B) At pH 4 in unbuffered solvent (green) and with 20 mM NaAc buffer (lightgreen), the most notable differences in α-helical content occur between residues 82-92. These residues correspond to helix F in holo-myoglobin. Interestingly, addition of NaAc buffer seems to lead to higher α-helical content in Helix F and reductions in most other sections of the protein. Compared to simulations at pH6, the α-helical content is most reduced in helices B,C,D, and in parts of helices E and G. C) Simulations at pH 2 do not fully unfold and show high averaged α-helical content for several residues. Helices H, G and parts of helices A and E retain a moderate to high helical content, with stronger decreases in helicality in helices B, C and D, when compared to simulations at pH 6 and 4. Interestingly, Parts of helix F seem to be especially stabilized at pH 2, in contrats to pH 4 and 6.

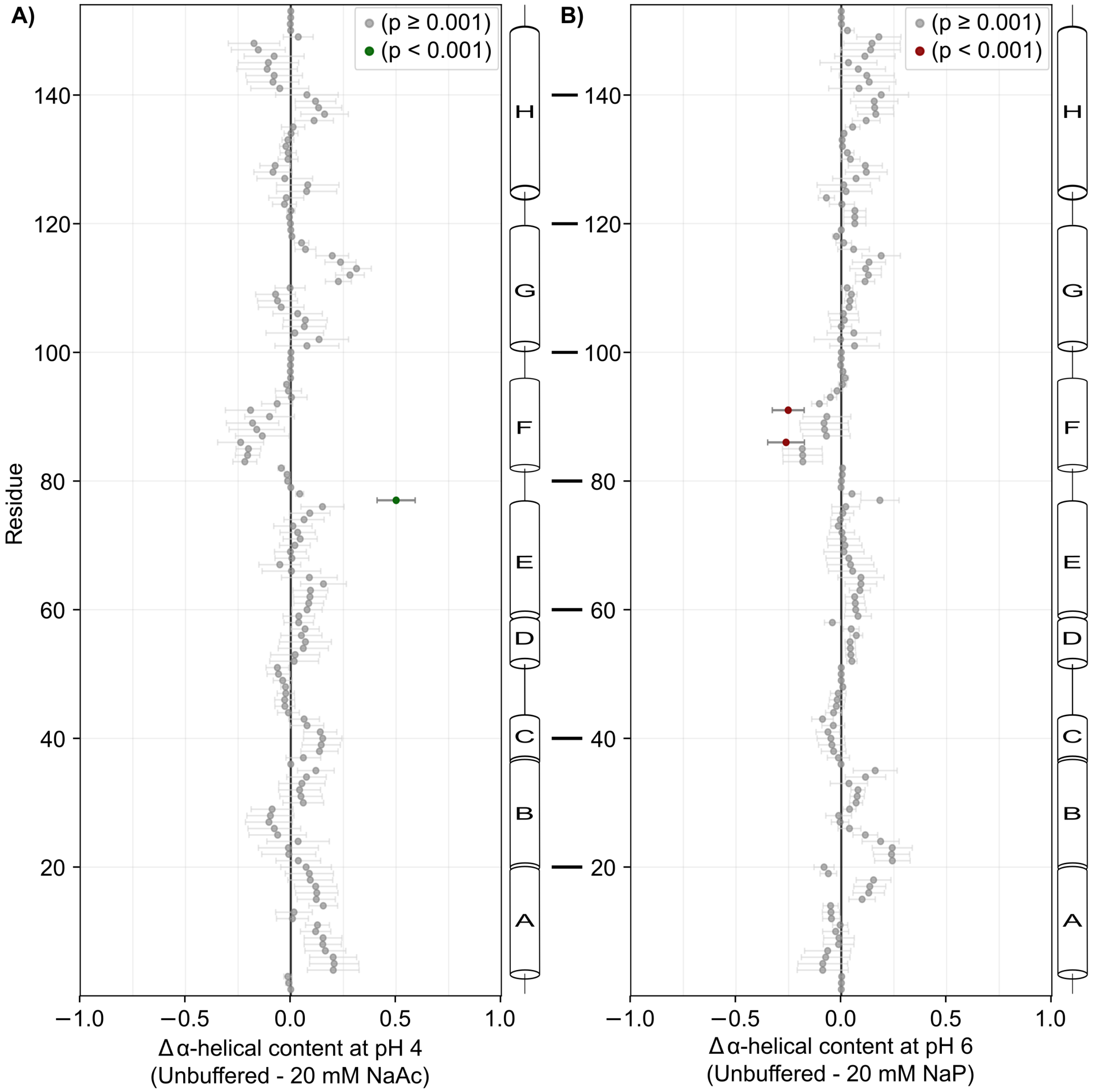

Figure S10: Difference in α-helical content per residue between unbuffered simulations and systems with either 20 mM NaAc or NaP buffer. For every residue of Apo-Mb, the averaged α-helical fraction $\pm$ sem (see Figure S9) of simulations with 20 mM NaAc buffer (A) or NaP buffer (B) was subtracted from the unbuffered simulations at pH 4 and 6, respectively. The colored dots signify mean values with a significant difference to the means of the whole data distribution, calculated via a T-test with a p-value cut-off of 0.001. Helix names and positions are represented to the right of each plot. In both cases, we find mostly small, statistically non-significant differences, with the exception of residues leucine 86 and glutamine 91 with NaP buffer at pH 6 (highlighted in red) or lysine 77 with NaAc buffer at pH 4 (highlighted in green).

S10 Simulation setup parameters.

All molecular dynamics simulations were prepared according to the parameters listed in Table S5.

Table S5: Simulation box setup.

Solvent condition	pH 6	pH 4	pH 2	pH 6 + 20 mM NaP	pH 4 + 20 mM NaAc
Box dimensions [Å] (x,y,z)[a)]	x = 83.206<br>y = 83.206<br>z = 83.206	x = 84.461<br>y = 84.461<br>z = 84.461	x = 83.228<br>y = 83.228<br>z = 83.228	x = 110.698<br>y = 111.421<br>z = 110.288	x = 110.857<br>y = 111.255<br>z = 110.329
Box angles (α, β, γ)[b)]	α = 109.471<br>β = 109.471<br>γ = 109.471<br>(truncated octahedron)	α = 109.471<br>β = 109.471<br>γ = 109.471<br>(truncated octahedron)	α = 109.471<br>β = 109.471<br>γ = 109.471<br>(truncated octahedron)	α = 90<br>β = 90<br>γ = 90<br>(cubic)	α = 90<br>β = 90<br>γ = 90<br>(cubic)
Number of solvent molecules [c)]	13883 H_2O<br>45 Cl^-<br>34 Na^+	13883 H_2O<br>45 Cl^-<br>34 Na^+	13883 H_2O<br>45 Cl^-<br>34 Na^+	43969 H_2O<br>14 $H_2PO_4^-$<br>115 Cl^-<br>118 Na^+	43976 H_2O<br>12 CH_3COOH<br>2 CH_3COO^-<br>127 Cl^-<br>118 Na^+
Number of replicas	11	11	11	11	11

a) After equilibration of box dimensions (as described in the methods section).
b) To optimize simulation performance, the simulation box volume was minimized using truncated octahedral boxes for all unbuffered simulations. As simulation preparation using PACKMOL -Memgen currently does not support truncated octahedral boxes, simulations with NaP or NaAc buffer were performed using cubic boxes.
c) As all titratable residues are set to the same, predefined protonation state during simulation setup, the same number of neutralizing ions was used for the equilibration steps of all unbuffered simulations. No protonation changes were performed during equilibration steps, only during production runs.

S11 Predicted pKa values across simulation conditions.

Protein residue pKa values were estimated assuming ideal Henderson-Hasselbalch behavior following the equation:

$$pKa = pH - log([A^{-}]/[HA]) \tag{S5}$$

Here, A^{-} and HA represent the de-protonated and protonated states of the target amino acid over the course of the simulation. This estimation does not consider potential effects arising from neighboring, interacting residues. However, more accurate pKa estimates would require performing extensive additional CpHMD simulations over a range of discrete pH values around the estimated residue pKa to allow fitting of the Hill equation to the deprotonated fraction over the range of simulated pH values. Furthermore, please note that the pKa values depend on the conformational state of the protein, as the electrostatic environment may change during conformational changes.

Table S6: Estimated residue pKa values.

	pH 6		pH 6 + 20 mM NaP		pH 4		pH 4 + 20 mM NaAc		pH 2	
Residue	Frac. Prot.	Pred. pKa	Frac. Prot.	Pred. pKa	Frac. Prot.	Pred. pKa	Frac. Prot.	Pred. pKa	Frac. Prot.	Pred. pKa
ASP 4	0.00 (0.01)	3.65 (0.54)	0.00 (0.00)	-	0.04 (0.05)	2.61 (0.54)	0.14 (0.10)	3.21 (0.37)	0.68 (0.25)	2.32 (0.50)
GLU 6	0.10 (0.13)	5.04 (0.65)	0.04 (0.03)	4.64 (0.32)	0.51 (0.22)	4.03 (0.38)	0.50 (0.28)	4.01 (0.48)	0.96 (0.06)	3.33 (0.65)
LYS 16	1.00 (0.00)	-	0.99 (0.01)	8.27 (0.63)	1.00 (0.00)	-	1.00 (0.00)	-	1.00 (0.00)	-
GLU 18	0.04 (0.03)	4.60 (0.35)	0.15 (0.15)	5.25 (0.50)	0.69 (0.27)	4.35 (0.55)	0.79 (0.25)	4.57 (0.66)	0.99 (0.02)	3.94 (0.59)
ASP 20	0.04 (0.02)	4.60 (0.21)	0.02 (0.02)	4.38 (0.45)	0.40 (0.18)	3.83 (0.32)	0.20 (0.18)	3.39 (0.50)	0.79 (0.25)	2.58 (0.65)
HIS 24	0.45 (0.26)	5.92 (0.46)	0.72 (0.20)	6.41 (0.43)	0.97 (0.02)	5.55 (0.30)	0.97 (0.04)	5.46 (0.58)	1.00 (0.00)	-
GLU 27	0.08 (0.06)	4.91 (0.36)	0.04 (0.02)	4.63 (0.27)	0.39 (0.16)	3.81 (0.29)	0.45 (0.29)	3.91 (0.51)	0.94 (0.05)	3.20 (0.40)
HIS 36	0.94 (0.10)	7.21 (0.77)	0.66 (0.32)	6.30 (0.63)	0.97 (0.03)	5.53 (0.45)	0.94 (0.08)	5.18 (0.61)	1.00 (0.00)	-
GLU 38	0.05 (0.07)	4.75 (0.62)	0.05 (0.03)	4.75 (0.28)	0.50 (0.22)	4.00 (0.38)	0.59 (0.25)	4.15 (0.45)	0.97 (0.02)	3.47 (0.31)
GLU 41	0.06 (0.04)	4.79 (0.34)	0.08 (0.05)	4.91 (0.29)	0.64 (0.26)	4.25 (0.49)	0.67 (0.28)	4.30 (0.55)	0.97 (0.02)	3.52 (0.24)
LYS 42	1.00 (0.00)	-	0.98 (0.06)	7.71 (1.34)	1.00 (0.00)	7.74 (1.44)	1.00 (0.00)	-	1.00 (0.00)	-
ASP 44	0.03 (0.09)	4.49 (1.32)	0.01 (0.02)	4.08 (0.56)	0.22 (0.29)	3.45 (0.75)	0.09 (0.11)	2.99 (0.59)	0.79 (0.14)	2.58 (0.37)
LYS 45	1.00 (0.00)	-	1.00 (0.00)	-	1.00 (0.00)	-	1.00 (0.00)	7.44 (1.10)	1.00 (0.00)	-
LYS 47	1.00 (0.01)	8.56 (0.83)	1.00 (0.00)	-	1.00 (0.00)	-	1.00 (0.00)	8.04 (1.44)	1.00 (0.00)	-
HIS 48	0.37 (0.20)	5.77 (0.38)	0.55 (0.22)	6.08 (0.38)	0.96 (0.02)	5.43 (0.20)	0.97 (0.02)	5.48 (0.29)	1.00 (0.00)	-
LYS 50	1.00 (0.00)	-	1.00 (0.00)	-	1.00 (0.00)	-	1.00 (0.00)	7.74 (1.44)	1.00 (0.00)	-
GLU 52	0.03 (0.01)	4.43 (0.23)	0.03 (0.02)	4.50 (0.29)	0.58 (0.24)	4.14 (0.43)	0.59 (0.25)	4.16 (0.46)	0.97 (0.03)	3.48 (0.43)
GLU 54	0.06 (0.03)	4.83 (0.19)	0.06 (0.02)	4.78 (0.20)	0.60 (0.14)	4.18 (0.25)	0.77 (0.10)	4.52 (0.24)	0.97 (0.03)	3.58 (0.45)
LYS 56	1.00 (0.01)	8.51 (0.98)	1.00 (0.00)	-	1.00 (0.00)	-	1.00 (0.00)	7.74 (0.97)	1.00 (0.00)	-
GLU 59	0.01 (0.00)	4.12 (0.08)	0.01 (0.01)	4.07 (0.22)	0.26 (0.08)	3.55 (0.17)	0.38 (0.19)	3.78 (0.35)	0.93 (0.05)	3.12 (0.34)
ASP 60	0.01 (0.02)	4.12 (0.80)	0.01 (0.01)	3.72 (0.44)	0.04 (0.05)	2.61 (0.59)	0.04 (0.06)	2.60 (0.71)	0.38 (0.34)	1.79 (0.63)
LYS 62	1.00 (0.00)	-	0.99 (0.01)	8.25 (0.48)	1.00 (0.00)	-	1.00 (0.00)	6.81 (0.44)	1.00 (0.00)	-
LYS 63	1.00 (0.00)	-	1.00 (0.00)	-	1.00 (0.00)	-	1.00 (0.00)	13.00 (0.00)	1.00 (0.00)	-
HIS 64	0.51 (0.23)	6.01 (0.39)	0.47 (0.25)	5.95 (0.44)	0.97 (0.04)	5.45 (0.59)	0.98 (0.02)	5.61 (0.34)	1.00 (0.00)	-
LYS 77	1.00 (0.00)	-	1.00 (0.00)	-	1.00 (0.00)	-	1.00 (0.00)	7.09 (0.46)	1.00 (0.00)	-
LYS 78	0.99 (0.01)	8.23 (1.06)	1.00 (0.00)	-	1.00 (0.00)	-	1.00 (0.00)	6.79 (0.84)	1.00 (0.00)	-
LYS 79	1.00 (0.00)	-	1.00 (0.00)	-	1.00 (0.00)	-	-	7.74 (1.44)	1.00 (0.00)	-
HIS 81	0.57 (0.19)	6.13 (0.34)	0.50 (0.19)	6.00 (0.33)	0.96 (0.05)	5.38 (0.59)	0.92 (0.08)	5.09 (0.50)	1.00 (0.00)	-
HIS 82	0.43 (0.34)	5.87 (0.60)	0.53 (0.34)	6.05 (0.60)	0.89 (0.14)	4.91 (0.63)	0.89 (0.19)	4.92 (0.85)	1.00 (0.00)	-
GLU 83	0.05 (0.03)	4.69 (0.31)	0.05 (0.03)	4.71 (0.28)	0.47 (0.17)	3.95 (0.29)	0.35 (0.17)	3.73 (0.32)	0.95 (0.04)	3.29 (0.40)
GLU 85	0.03 (0.03)	4.51 (0.38)	0.05 (0.03)	4.74 (0.26)	0.47 (0.22)	3.95 (0.39)	0.57 (0.23)	4.12 (0.42)	0.96 (0.03)	3.42 (0.38)
LYS 87	1.00 (0.00)	-	1.00 (0.00)	-	1.00 (0.00)	-	1.00 (0.00)	-	1.00 (0.00)	-
HIS 93	0.61 (0.21)	6.19 (0.38)	0.47 (0.25)	5.95 (0.43)	0.96 (0.05)	5.42 (0.62)	0.98 (0.02)	5.61 (0.36)	1.00 (0.00)	-
LYS 96	1.00 (0.00)	-	1.00 (0.00)	-	1.00 (0.00)	-	1.00 (0.00)	-	1.00 (0.00)	-
HIS 97	0.36 (0.21)	5.75 (0.40)	0.49 (0.23)	5.98 (0.40)	0.97 (0.03)	5.48 (0.41)	0.97 (0.02)	5.59 (0.27)	1.00 (0.00)	-
LYS 98	1.00 (0.00)	-	1.00 (0.00)	-	1.00 (0.00)	-	1.00 (0.00)	-	1.00 (0.00)	-
LYS 102	1.00 (0.00)	-	1.00 (0.00)	-	1.00 (0.00)	-	1.00 (0.00)	-	1.00 (0.00)	-
TYR 103	1.00 (0.00)	-	1.00 (0.00)	-	1.00 (0.00)	-	1.00 (0.00)	-	1.00 (0.00)	-
GLU 105	0.03 (0.02)	4.46 (0.37)	0.04 (0.03)	4.62 (0.31)	0.61 (0.28)	4.20 (0.52)	0.51 (0.32)	4.02 (0.56)	0.99 (0.01)	4.09 (0.38)
ASP 109	0.00 (0.00)	-	0.00 (0.00)	-	0.03 (0.03)	2.44 (0.44)	0.04 (0.04)	2.58 (0.54)	0.56 (0.35)	2.11 (0.62)
HIS 113	0.45 (0.26)	5.91 (0.45)	0.66 (0.25)	6.29 (0.48)	0.96 (0.08)	5.36 (0.84)	0.98 (0.02)	5.73 (0.37)	1.00 (0.01)	4.31 (0.55)
HIS 116	0.52 (0.21)	6.04 (0.36)	0.51 (0.11)	6.03 (0.20)	0.93 (0.15)	5.10 (0.93)	0.90 (0.17)	4.95 (0.80)	1.00 (0.01)	4.31 (0.52)
LYS 118	1.00 (0.00)	-	1.00 (0.00)	-	1.00 (0.00)	-	1.00 (0.00)	-	1.00 (0.00)	-
HIS 119	0.67 (0.24)	6.31 (0.46)	0.72 (0.09)	6.40 (0.20)	0.98 (0.01)	5.76 (0.29)	0.98 (0.01)	5.76 (0.32)	1.00 (0.00)	-
ASP 122	0.01 (0.01)	4.17 (0.24)	0.01 (0.02)	4.16 (0.49)	0.24 (0.16)	3.50 (0.37)	0.25 (0.17)	3.52 (0.39)	0.91 (0.07)	3.01 (0.35)
ASP 126	0.01 (0.00)	-	0.01 (0.01)	4.05 (0.54)	0.26 (0.27)	3.55 (0.61)	0.16 (0.11)	3.29 (0.33)	0.87 (0.23)	2.82 (0.86)
LYS 133	1.00 (0.00)	-	0.99 (0.01)	8.26 (0.42)	1.00 (0.00)	-	1.00 (0.00)	-	1.00 (0.00)	-
GLU 136	0.04 (0.04)	4.62 (0.45)	0.04 (0.02)	4.64 (0.25)	0.46 (0.14)	3.94 (0.24)	0.45 (0.16)	3.92 (0.28)	0.92 (0.06)	3.05 (0.33)
ASP 141	0.11 (0.19)	5.10 (0.83)	0.02 (0.03)	4.26 (0.64)	0.18 (0.22)	3.35 (0.65)	0.26 (0.22)	3.55 (0.49)	0.57 (0.38)	2.13 (0.67)
LYS 145	0.99 (0.01)	8.01 (0.44)	1.00 (0.00)	-	1.00 (0.00)	-	1.00 (0.01)	6.51 (1.12)	1.00 (0.00)	-
TYR 146	1.00 (0.00)	-	1.00 (0.00)	-	1.00 (0.00)	-	1.00 (0.00)	-	1.00 (0.00)	-
LYS 147	1.00 (0.00)	-	1.00 (0.00)	-	1.00 (0.00)	-	1.00 (0.00)	-	1.00 (0.00)	-
GLU 148	0.12 (0.12)	5.13 (0.50)	0.09 (0.05)	5.02 (0.26)	0.62 (0.23)	4.22 (0.43)	0.64 (0.12)	4.25 (0.22)	0.99 (0.01)	-

S12 RMS average correlation analysis

The RMS average correlation (RAC) [25] was calculated and averaged over all simulation replicas to assess the convergence of the sampled protein conformations.

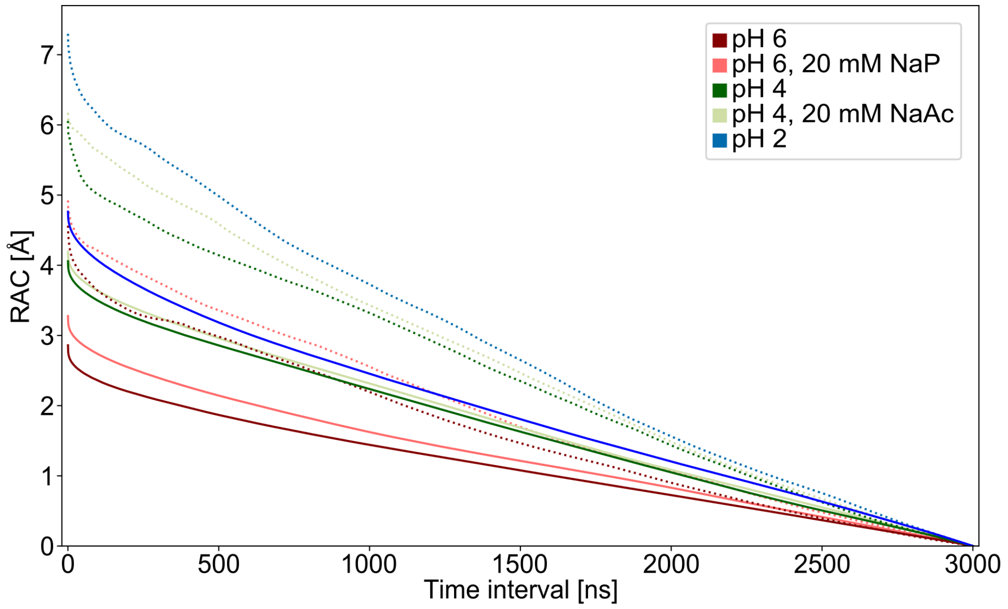

Figure S11: RMS average correlation (RAC) curves for Cα atoms of Apo-Mb in unbuffered solvent conditions at pH 6 (dark red), pH 4 (dark green), pH 2 (blue), with 20 mM NaP buffer at pH 6 (light red) or with 20 mM NaAc buffer at pH 4 (light green). Simulation frames were analyzed with an offset of 200 ps. Atoms of each simulation frame were superimposed to either the first frame (dotted lines) or the average structure over all frames (solid lines).

All simulations performed in this work start from the same initial structure resembling the Holo-Mb crystal structure (PDB-ID: 2V1K). In the different solvent conditions, the protein unfolds increasingly the lower the pH gets or upon the addition of either 20 mM NaP or NaAc buffer. Even for the solvent conditions with the least unfolding (pH 6 unbuffered and pH 6 with 20 mM NaP buffer), no clear cut-off timeframe for conformational convergence can be determined from the RAC plots above. Still, the generally smooth curves indicate that the systems explore their conformational spaces in a relatively continuous and consistent way, rather than jumping abruptly between unrelated states.

S13 Cluster discovery analysis over the last 500 ns of each simulation

All frames of the last 500 ns of each simulation replica were clustered using hierarchical agglomerative clustering based on the protein backbone RMSD as a cluster distance metric with a minimum cluster distance of 3 Å. To compare the diversity in conformational samples, the sum of new clusters discovered over the last 500 ns was plotted for every replica together with the averaged values [22]. While the α-helical content of Apo-Mb is stable over the last 500 ns of simulations at pH 6 and 4 with and without additional buffers (see SI Figure S14 below), the number of discovered clusters roughly converges across all replicas for simulations at pH 6 only. Addition of NaP buffer and lowering of the pH leads to some simulation replica exploring novel conformations even at the later stages of the simulations. These observations can be mainly attributed to the technical difficulties of assigning discrete conformations to a protein consisting of around 50% flexible loop regions at pH 6, which further increases to ~60-70% loops at pH 4 and even further at pH 2.

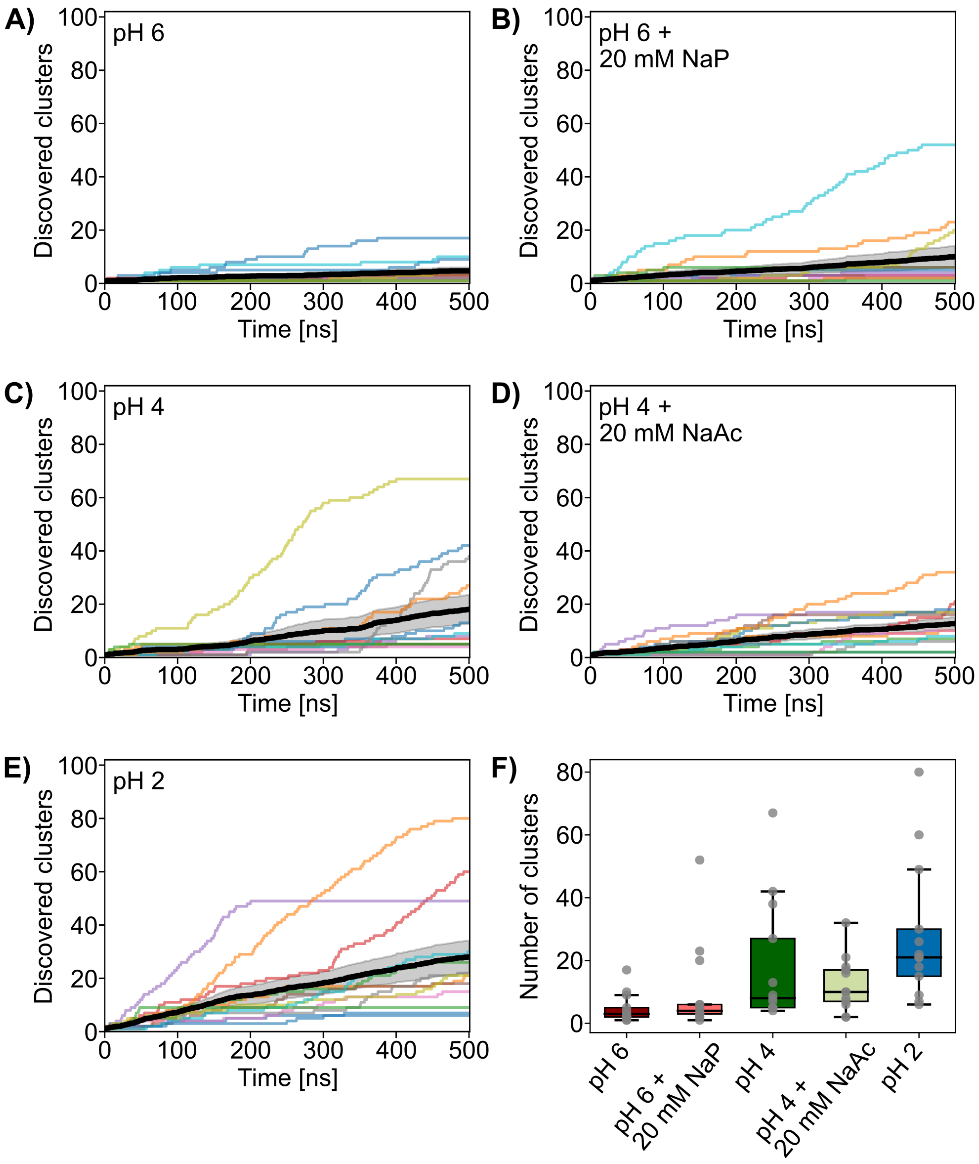

Figure S12: Cluster discovery analysis for the last 500 ns of each simulation. (A,C,E) Cumulative sum of protein structure clusters over the simulation time for solvent conditions at pH 6, 4, and 2, as well as (B) at pH 6 with additional 20 mM NaP buffer and (D) at pH 4 with additional 20 mM NaAc buffer. The number of clusters for every replica are colored, while the averaged value is plotted in black with the standard deviation highlighted in gray. (F) Total sum of clusteres discovered for every individual replica in simulations at pH 6 without and with 20 mM NaP buffer (dark red and light red), at pH 4 without and with 20 mM NaAc buffer (dark green and light green), and at pH 2 (blue). Boxplots indicate the distribution over the replicas with gray dots highlighting each individual value.

S14 Cluster discovery analysis over the full simulation time

To assess the overall conformational diversity over the performed unfolding simulations, every fifth frame (500 ps time interval) of each simulation replica was clustered using the same hierarchical agglomerative clustering approach as in Figure S12 [22].

Simulations at pH 6 form the lowest number of clusters over time and also show the smallest deviations between simulation replicas. The addition of NaP buffer and lowering of the pH increases both the total number of clusters and the divergence between the behavior of the individual simulation replicas. Interestingly, even at pH 2, two replicas explore only a low number of clusters, indicating that the time of the unfolding process of Apo-Mb at this pH value exceeds the simulation time of 3 μs, in line with previous reports [26, 27]

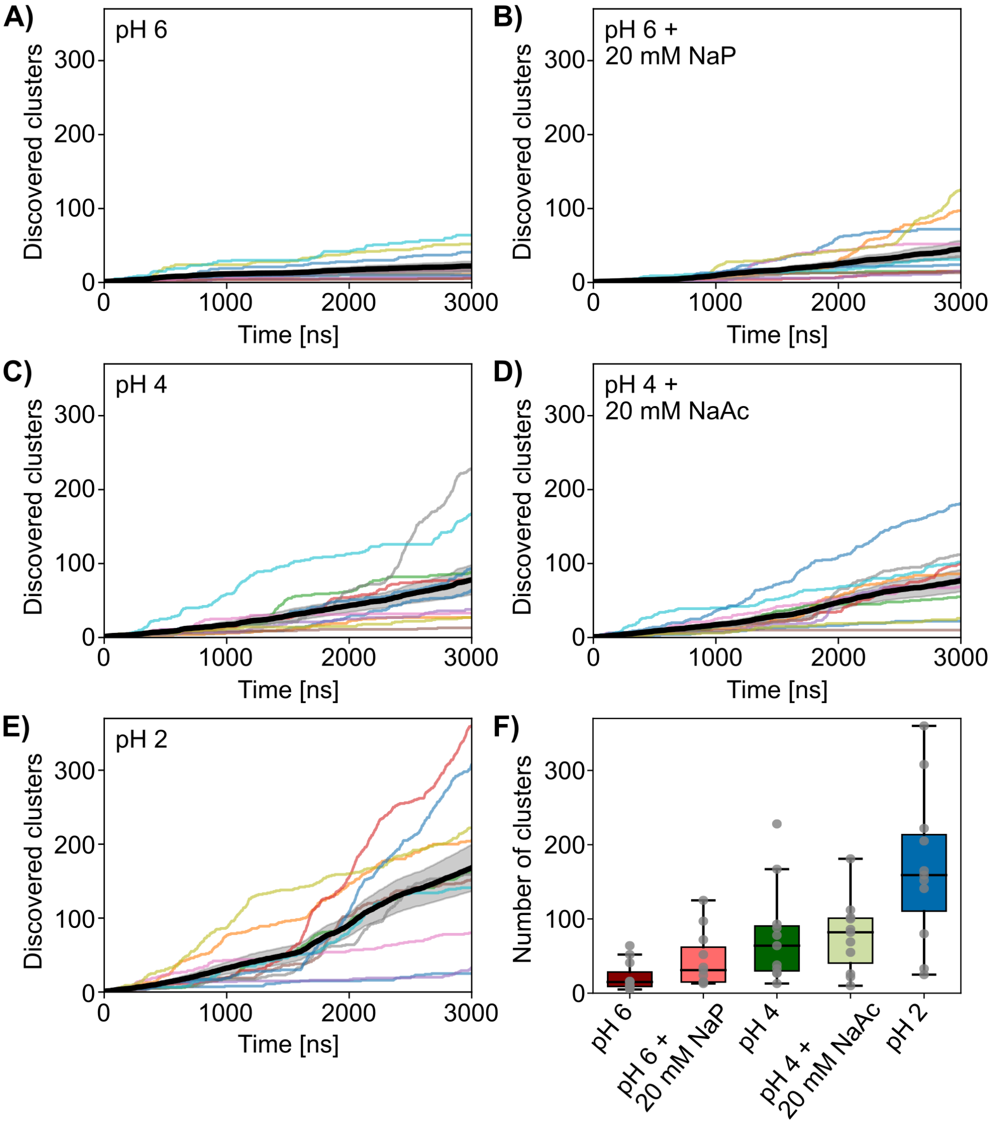

Figure S13: Cluster discovery analysis for the full length of each simulation replica. (A,C,E) Cumulative sum of protein structure clusters over the full simulation time for solvent conditions at pH 6, 4, and 2, as well as (B) at pH 6 with additional 20 mM NaP buffer and (D) at pH 4 with additional 20 mM NaAc buffer. The numbers of clusters for every replica are colored, while the averaged value is plotted in black with the standard deviation highlighted in gray. (F) Total sum of clusters discovered for every individual replica in simulations at pH 6 without and with 20 mM NaP buffer (dark red and light red), at pH 4 without and with 20 mM NaAc buffer (dark green and light green) and at pH 2 (blue). Boxplots indicate the distribution over the replicas with gray dots highlighting each value.

S15 Convergence of the average α-helical content per simulation condition

As the helical content of Apo-Mb was used as the experimental measure for assessing the folding state of Apo-Mb (see Table 1 in the main text), we selected simulation frames with a similar α-helical content for analysis for every solvent condition. This resulted in the last 500 ns of simulation time being selected as a timeframe with low overall change in helicality values (slope of the linear fit is close to zero).

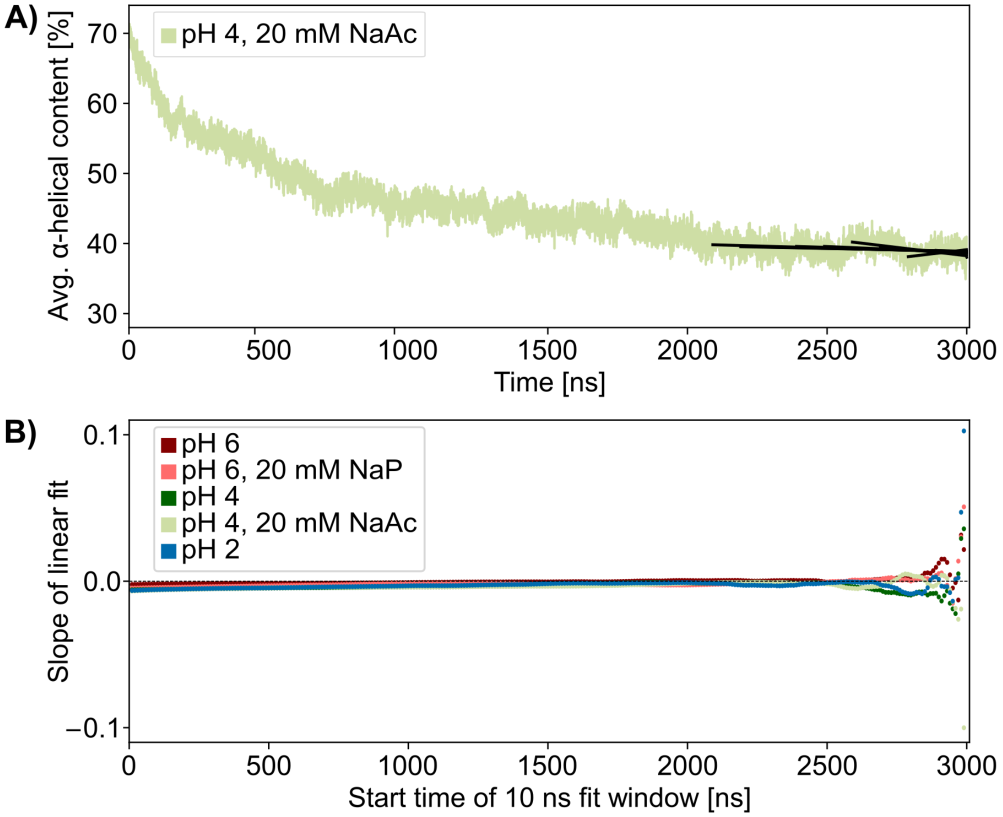

Figure S14: Convergence of the average α-helical content over all simulation conditions estimated by linear fits with decreasing window sizes in 10 ns steps. A) The average α-helical content of Apo-Mb in simulation conditions at pH 4 with 20 mM of NaAc buffer (light green) with exemplary linear fits (black) with decreasing window size. The plotted lines represent linear fits over ten example windows. B) Slopes of the fitted lines plotted at the starting time of the respective window, i.e. a linear fit from 2100 - 3000 ns is represented by a dot at 2100 ns.

While the slopes stay close to zero for most of the five simulated solvent conditions, the inherent noise due to fewer sampled data leads to a noticeable increase in slopes towards the end of the simulation time. We, therefore, selected the last 500 ns window for further analysis steps as a compromise between Apo-Mb unfolding and conformational diversity at the targeted α-helical content.

S16 Distribution of water molecules in the 1st and 2nd water shells

The number of water molecules around the protein in the 1st and 2nd hydration shell was determined for the last 500 ns of all replicas for all simulated solvent conditions: The overall number of water molecules increases at lower pH levels and upon the addition of either NaP or NaAc buffer. Lower pH values and buffer addition also lead to broader shoulders in the histograms and, in the case of NaP buffer at pH 6, to the formation of an additional, right-shifted histogram peak. These changes in the data distributions potentially stem from the increased diversity in conformations at lower pH values and upon buffer addition (compare Figure 2 in the main text and Figures S12 & S13 above).

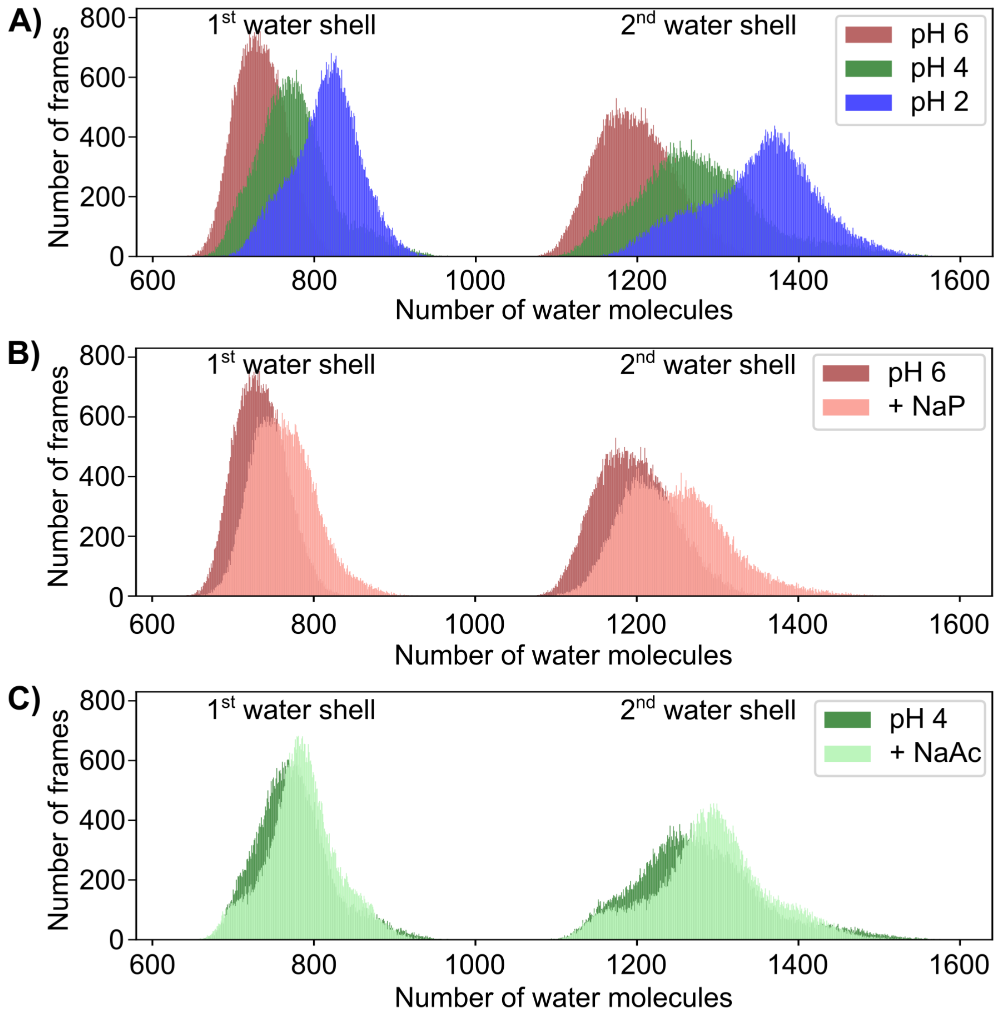

Figure S15: Number of water molecules in the 1st and 2nd water shell around Apo-Mb, comparing solvent conditions at (A) pH 2, 4, and 6, (B) at pH 6 with and without 20 mM of NaP buffer and (C) at pH 4 with and without 20 mM of NaAc buffer. The cut-offs for the first and second water shells around the protein are 3.4 Å and 5.0 Å, respectively.

References

[1] M. Kataoka, I. Nishii, T. Fujisawa, T. Ueki, F. Tokunaga, and Y. Goto. Structural characterization of the molten globule and native states of apomyoglobin by solution x-ray scattering. *J. Mol. Biol.*, 249:215–228, 1995.

[2] M. Doi and S. F. Edwards. *The theory of polymer dynamics*, volume 73 of *International series of monographs on physics*. Clarendon Press, Oxford, reprint edition, 2013.

[3] R. J. Hunter. *Zeta Potential in Colloid Science*. Academic Press, 1981.

[4] F. Wang and X.-J. Tang. Conformational heterogeneity and stability of apomyoglobin studied by hydrogen/deuterium exchange and electrospray ionization mass spectrometry. *Biochem.*, 35:4069–4078, 1996.

[5] Y. Goto and A. L. Fink. Phase diagram for acidic conformational states of apomyoglobin. *J. Mol. Biol.*, 214:803–805, 1990.

[6] T. Mizukami, Y. Sakuma, and K. Maki. Statistical mechanical model for ph-induced protein folding: Application to apomyoglobin. *J . Phys. Chem. B*, 120:8970–8986, 2016.

[7] L. Balacescu, T. E. Schrader, A. Radulescu, P. Zolnierczuk, O. Holderer, St. Pasini, J. Fitter, and A. M. Stadler. Transition between protein-like and polymer-like dynamic behavior: Internal friction in unfolded apomyoglobin depends on denaturing conditions. *Sci. Rep.*, 10:1570, 2020.

[8] L. Konermann and D. J. Douglas. Equilibrium unfolding of proteins monitored by electrospray ionization mass spectrometry: distinguishing two-state from multi-state transitions. *Rapid Commun. Mass Spectrom.*, 12:435–442, 1998.

[9] G. Wittko and W. Köhler. Precise determination of the Soret, thermal diffusion and mass diffusion coefficients of binary mixtures of dodecane, isobutylbenzene and 1,2,3,4-tetrahydronaphthalene by a holographic grating technique. *Philos. Mag.*, 83:1973–1987, 2003.

[10] Y. V. Griko, P. L. Privalov, S. Y. Venyaminov, and V. P. Kutyshenko. Thermodynamic study of the apomyoglobin structure. *J. Mol. Biol.*, 202:127–138, 1988.

[11] Z. Wang, H. Kriegs, and S. Wiegand. Thermal diffusion of nucleotides. *J. Phys. Chem. B*, 116:7463–7469, 2012.

[12] D. Kameoka, E. Masuzaki, T. Ueda, and T. Imoto. Effect of buffer species on the unfolding and the aggregation of humanized igg. *J. Biochem.*, 142:383–391, 2007.

[13] S. H. Kim, H. J. Yoo, E. J. Park, and D. H. Na. Nano differential scanning fluorimetry-based thermal stability screening and optimal buffer selection for immunoglobulin g. *Pharmaceuticals*, 15:29, 2022.

[14] V. N. Viswanadhan, A. K. Ghose, Ganapathi R. Revankar, and R. K. Robins. Atomic physicochemical parameters for three dimensional structure directed quantitative structure-activity relationships. 4. additional parameters for hydrophobic and dispersive interactions and their

application for an automated superposition of certain naturally occurring nucleoside antibiotics. *J. Chem. Inf. Model.*, 29:163–172, 1989.

[15] A. Eiberweiser. *Hydration and Ion Pairing of Aqueous Phosphate Solutions as Observed by Dielectric Spectroscopy.* PhD thesis, Universität, Regensburg, 2013.

[16] L. Medda, C. Carucci, D. F. Parsons, B. W. Ninham, M. Monduzzi, and A. Salis. Specific cation effects on hemoglobin aggregation below and at physiological salt concentration. *Langmuir*, 29:15350–15358, 2013.

[17] H. M. A. Rahman, G. Hefter, and R. Buchner. Hydration of formate and acetate ions by dielectric relaxation spectroscopy. *J. Phys. Chem. B*, 116:314–323, 2012.

[18] S. Y. Willow and S. S. Xantheas. Molecular-level insight of the effect of Hofmeister anions on the interfacial surface tension of a model protein. *J. Phys. Chem. Lett.*, 8:1574–1577, 2017.

[19] C. N. Pace, H. Fu, K. L. Fryar, J. Landua, S. R. Trevino, B. A. Shirley, M. M. Hendricks, S. Iimura, K. Gajiwala, J. M. Scholtz, and G. R. Grimsley. Contribution of hydrophobic interactions to protein stability. *J. Mol. Biol.*, 408:514–528, 2011.

[20] D. Niether and S. Wiegand. Thermophoresis of biological and biocompatible compounds in aqueous solution. *J. Phys. Condens. Matter*, 31:503003, 2019.

[21] David A. Case, Hasan Metin Aktulga, Kellon Belfon, David S. Cerutti, G. Andrés Cisneros, Vinícius Wilian D. Cruzeiro, Negin Forouzesh, Timothy J. Giese, Andreas W. Götz, Holger Gohlke, Saeed Izadi, Koushik Kasavajhala, Mehmet C. Kaymak, Edward King, Tom Kurtzman, Tai-Sung Lee, Pengfei Li, Jian Liu, Tyler Luchko, Ray Luo, Madushanka Manathunga, Matias R. Machado, Hai Minh Nguyen, Kurt A. O'Hearn, Alexey V. Onufriev, Feng Pan, Sergio Pantano, Ruxi Qi, Ali Rahnamoun, Ali Risheh, Stephan Schott-Verdugo, Akhil Shajan, Jason Swails, Junmei Wang, Haixin Wei, Xiongwu Wu, Yongxian Wu, Shi Zhang, Shiji Zhao, Qiang Zhu, Thomas E. III Cheatham, Daniel R. Roe, Adrian Roitberg, Carlos Simmerling, Darrin M. York, Maria C. Nagan, and Kenneth M. Jr. Merz. AmberTools. *J. Chem. Inf. Model.*, 63:6183–6191, 2023.

[22] Roe, D. R. and Cheatham, T. E. III. PTRAJ and CPPTRAJ: Software for Processing and Analysis of Molecular Dynamics Trajectory Data. *J. Chem. Theory Comput.*, 9:3084–3095, 2013.

[23] Case, D.A., Aktulga, H.M., Belfon, K., Ben-Shalom, I.Y., Berryman, J.T., Brozell, S.R., Carvahol, F.S., Cerutti, D.S., Cheatham, T.E., III, Cisneros, G.A., Cruzeiro, V.W.D., Darden, T.A., Forouzesh, N., Ghazimirsaeed, M., Giambaşu, G., Giese, T., Gilson, M.K., Gohlke, H., Goetz, A.W., Harris, J., Huang, Z., Izadi, S., Izmailov, S.A., Kasavajhala, K., Kaymak, M.C., Kolossváry, I., Kovalenko, A., Kurtzman, T., Lee, T.S., Li, P., Li, Z., Lin, C., Liu, J., Luchko, T., Luo, R., Machado, M., Manathunga, M., Merz, K.M., Miao, Y., Mikhailovskii, O., Monard, G., Nguyen, H., O'Hearn, K.A., Onufriev, A., Pan, F., Pantano, S., Rahnamoun, A., Roe, D.R., Roitberg, A., Sagui, C., Schott-Verdugo, S., Shajan, A., Shen, J., Simmerling, C.L., Skrynnikov, N.R., Smith, J., Swails, J., Walker, R.C., Wang, J., Wang, J., Wu, X., Wu, Y., Xiong,

Y., Xue, Y., York, D.M., Zhao, C., Zhu, Q., Kollman, P.A. (2024), Amber 2024, University of California, San Francisco.

[24] P. Picotti, A. Marabotti, A. Negro, V. Musi, B. Spolaore, M. Zambonin, and A. Fontana. Modulation of the structural integrity of helix f in apomyoglobin by single amino acid replacements. *Protein Sci.*, 13:1572–1585, 2004.

[25] R. Galindo-Murillo, D. R. Roe, and T. E. Cheatham. Convergence and reproducibility in molecular dynamics simulations of the dna duplex d(gcacgaacgaacgaacgc). *Biochim. Biophys. Acta Gen. Subj.*, 1850:1041–1058, 2015.

[26] M. Jamin, M. Antalik, S. N. Loh, D. W. Bolen, and R. L. Baldwin. The unfolding enthalpy of the ph 4 molten globule of apomyoglobin measured by isothermal titration calorimetry. *Protein Science*, 9:1340–1346, 2000.

[27] M. Jamin, S. Yeh, D. L. Rousseau, and R. L. Baldwin. Submillisecond unfolding kinetics of apomyoglobin and its ph 4 intermediate. *J. Mol. Biol.*, 292:731–740, 1999.
